# A memorized multi-objective Sinh-Cosh optimizer for solving multi-objective engineering design problems

**DOI:** 10.1038/s41598-025-33789-8

**Published:** 2026-01-21

**Authors:** Doaa El-Nagar, Ibrahim Zeidan, Mohamed Issa

**Affiliations:** 1 Computer and Systems Department, Faculty of Engineering, Zagazig University, Zagazig, Egypt; 2 Faculty of Computer Science and Information Technology , Egypt-Japan University for Science and Technology, Alexandria, Egypt

**Keywords:** Multi-objective optimization, Metaheuristic, Sinh-Cosh algorithm and memorized technique, Engineering, Mathematics and computing

## Abstract

The Multi-Objective Sinh-Cosh Optimization Algorithm (MOSCHO) is presented in this article based on the memorized technique. MOSCHO is an extension version of the recently proposed Sinh-Cosh optimizer for multiple objective optimizations. The memorized local optimum is integrated with the global optimal solution to bound the search space and update positions of solutions for obtaining non-dominated solutions. The proposed method is tested on mathematical non-constrained functions, SRN constrained function, and three real-world design engineering applications, as a vital challenge to handle the difficulties of real-world engineering applications. The MOSCHO’s performance was evaluated by seven performance metrics compared to some of the most popular multi-objective optimization algorithms. The results demonstrate the ability of MOSCHO to achieve a high convergence and a good diversity. The results clarify that three functions have the best performance for all tested performance metrics: ZDT3, ZDT4, and MMF14. Five functions have the best performance for more than 75% of the performance metrics. Two functions have the best performance for more than 50% of performance metrics. The others have only the best values for more than 25% of performance metrics. However, SRN and real-world problems exhibit the best performance in more than 75% of the tested performance metrics.

**Supplementary Information:**

The online version contains supplementary material available at 10.1038/s41598-025-33789-8.

## Introduction

For the last decades, meta-heuristic algorithms have been used more and more for more complex, non-linear, high-dimensional, large-scale problems. The exact algorithm achieves more accurate solutions due to coverage of the whole search space and higher consumption of processing time. But Meta-heuristic algorithms achieve an optimal solution for many complicated applications and consume less processing time for complex, high-dimensional, large-scale applications, especially more complicated engineering applications^1^.

Meta-heuristic optimization is the process of finding good solutions, where this solution is not necessarily an optimal solution, but mainly discards bad solutions. Meta-heuristic is used for a wide range of variant problems, such as engineering applications, medical applications, etc., because it has many variant algorithms that achieve good solutions, which are sufficient to be an optimal solution for many complex problems. With the vast computer-aided design, researchers and engineers can convert a real system to a computer model that contains its variables and constraints as a Simulink model concerned with system architecture, without the need to contain a real system or its prototype to test that real-world system. That leads to reduced human interactions and expensive methods of overall real systems or design models for manufacturing methods. Due to structural properties, constraints, ambiguous weights, operating conditions, manufacturing processing details, and all other system conditions, huge researchable problems as engineering problems. But predetermined optimization algorithms may lead to an inconsistent, inefficient, and design failure. So, many nondeterministic optimization algorithms, such as meta-heuristic algorithms, can solve many different design problems of real or manufacturing processes of various problems^2^.

Meta-heuristic optimization algorithm handles any application based on repetitive iterations with random solutions enhanced at each iteration to reach optimal solutions. During the iterative method, the meta-heuristic directs candidate solutions towards optimal solutions and allows these solutions to evade the portions of the search space that contain the local optimal solutions or optimal solutions that are not located in these portions of the search space. Meta-heuristic algorithms depend on methods that are inspired by nature, mathematical functions, and scientific rules that can avoid local optimal solutions, such as Particle Swarm Optimization (PSO)^3,4^, Salp Swarm Algorithm (SSA)^5^, Whale Optimization Algorithm (WOA), Dynamic Arithmetic optimization (DAO), MRBMO: An Enhanced Red-Billed Blue Magpie Optimization Algorithm for Solving Numerical Optimization Challenges^6^, Sinh Cosh Optimizer (SCO)^7^ and Artificial Lemming Algorithm^8^. Metaheuristic algorithms have proven highly effective in solving complex engineering optimization problems, with successful applications spanning diverse fields such as bioinformatics^9,10^, Sequence Alignment^10–15^, PID controller optimization^16–18^, solar energy systems^19–21^, Fuel Cell^20,21^ and passive suspension systems^22^. Consequently, these techniques have become a prominent approach for controller design.

Meta-heuristic methods use only one solution, which is used rarely for simpler applications or many solutions, called population-based, to look for optimal solutions that are more famous, effective, and realistic for various applications. They are used to find only one optimal solution, called a single objective optimization algorithm, or find multiple related solutions, called a multi-objective optimization algorithm. Single objective optimization has more ability to avoid local optimal solutions and consumes less processing time with a straightforward structure that is easy to implement and has very good precision. But it handles only one objective to find the maximum or minimum optimal solution. But multi-objective optimization handles simultaneously many conflicting objectives like maximizing efficiency, minimizing cost, and minimizing the impact on the environment. It has become useful and popular for many fields, like industrial, engineering, physical, economic, and medical applications.

Multi-objective optimization is considered a decision-making process that can balance multiple objectives simultaneously, but single-objective optimization is considered a part of multi-objective optimization that represents only one objective and focuses its process on that objective only, like a cost or any other objective concerned with the problem. Multi-objective optimization has gained a vast majority for many problems because there is a need to handle more complicated and multiple objective applications, especially over the last few decades^23^. The goal of the multi-objective optimization is to find multiple non-dominated optimal solutions called Pareto optimal solutions, in which improving each objective does not lead to deteriorating any other objective.

**Fig. 1:**
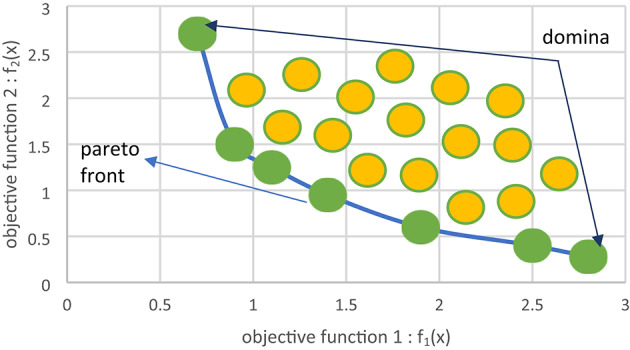
Pareto optimal solutions.

But it is hard to find exact Pareto-optimal solutions with an exact size for all applications. So, metaheuristics try to find an approximation or effective subset of solutions as a Pareto-optimal non-dominated set. A Pareto-optimal solution is a non-dominated solution that is formed by a solution that has increasing efficiency of at least one objective while maintaining the best efficiency reached of other objectives or has an increase in their efficiencies. That collection reached a Pareto optimal set or Pareto front set^24^, as in Fig. 1.

The general form of multi-objective optimization of any problem is expressed as follows in Eq. 1.

1 $$\:minimize\:F\left(x\right)=\:\left({f}_{1}\left(x\right),\:\:{f}_{2}\left(x\right),\:{f}_{3}\left(x\right),\dots\:\dots\:\dots\:\dots\:\dots\:,{f}_{n}\left(x\right)\:\:\right),\:\:\:x\:\in\:S$$

Where *n* ≥ 2 expresses the number of objectives, x ($\:{x}_{1}\:,\:{x}_{2},\dots\:\dots\:\dots\:..,{x}_{k}$), x represents the vector of problem decision variables, k represents dimension of decision variables, S represents decision space or search space of equality and inequality problem constrains for its boundaries, F(x) represents objective vector to be optimized through multi-objective optimization algorithm that is defined as a cost function of each objective solution where objective cost function is a continuous and constrained multivariable problem^25^.

No-Free-Lunch theorem (NFL) states that no algorithm or method is the best for solving all optimization problems of different natures and different data^26^. This principle is a good chance for improving existing algorithms or implementing new algorithms to obtain the best solution for a particular problem or find a reliable solution for unconstrained problems. So new algorithms may have an effect on finding the best applicable solution of one or many of the unconstrained and constrained optimization problems^27^.

There are major categories established to handle multi-objective optimization problems, such as the single-to-multi transformation approach, that based on aggregating many objectives in a single scalar form, non-dominated sorting methods, decomposition-based methods that are based on ranking solutions according to Pareto dominance, decomposition-based methods that are based on decomposing the problem into sub-problems, and indicator-based methods that are based on using performance indicators to guide selection.

While extending single-objective algorithms to handle multiple objectives simultaneously. Many techniques are established. The simple and early method is to aggregate objectives with fixed weights or optimize only one objective while considering the others as constraints. But the most reliable technique is adapting the swarm optimization to generate a distributed set of Pareto optimal solutions as multi-objective particle swarm optimization^28^.

The non-dominated sorting methods are proposed based on Pareto dominance according to crowding strategies to rank solutions as early non-dominated sorting genetic algorithm (NSGA), then the Pareto Archived Evolution Strategy (PEAS), then improving NSGA with a fast non-dominated sorting genetic algorithm and crowding distance that results in NSGAII^29^.

The decomposition-based methods in which problems are decomposed into a scalar subproblems. Handling each subproblem as a separate problem, then combining the results to obtain a problem solution. These methods are proposed as a Multi-Objective Evolutionary Algorithm based on Decomposition (MOEA/D), Weighted Tchebycheff Method, and Penalty Boundary Intersection (PBI)^30^.

Finally, indicator-based methods that use the performance indicators as hypervolume for the selection method as the indicator-Based Evolutionary Algorithm and the S-Metric Selection Evolutionary Multi-Objective Algorithm^30^.

There are many models that have been proposed by swarm algorithms or any evolutionary algorithms over the last years to reach a Pareto set to solve multi-objective optimization problems. Many algorithms are extended to solve multiple interfering objectives by finding a set of non-dominated solutions, such as the non-dominated sorting genetic algorithm (NSGAII)^24^. It is one of the multi-objective genetic algorithms. A Pareto Achieved Evolution Strategy (PEAS) was also one of the multi-objective genetic algorithms proposed to solve multi-objective optimization problems. Its process starts by generating initially many candidate solutions, then evaluates them by many objective functions to find archived non-dominated solutions. Multi-objective genetic algorithms focus on confining the search space precisely. So, they are reliable, effective, population-based approaches for many multi-objective problems^31^.

A Dynamic Multi-Objective Evolutionary Algorithm was another proposed model in which cell population size varies dynamically through its process. Its strategies depend on diversity and dominance data to estimate density and rank solutions as a qualitative parameter to decide whether to add or remove candidate solutions to the cell. Its diversity strategy leads to a well Pareto optimal front for many constraints, but according to the NFL theorem, it isn’t able to maintain all problem characteristics for many of the test problems^32^.

A distributed cooperative coevolutionary algorithm has a competitive advantage in that distributed sub-populations with a shared archive are processed on many computers within the same network to reduce running time^33^. For interval multi-objective problems, an evolutionary algorithm with a preference polyhedron is proposed, where the preference polyhedron and decision makers interact with each other to reach the best performance^30^. Varied subspaces of weighting vectors are extracted from objective space uniformly for A Decomposition-based achieving approach, in which Pareto dominance normalized distance-based is considered for accepting subspace solution or not. When accepting a non-dominated subspace solution, it is added to the shared diversity archive. The last approach speeds convergence and enhances diversity for various test functions used^34^. But other interval algorithms for various local searches generated for good convergence and better distribution of the Pareto optimal set proposed by Sun^35^.

Many subproblems with neighboring information processing simultaneously of the multi-objective problem are the main method of another decomposition algorithm, which is tested for many versions of the multi-objective genetic algorithm, such as the local search genetic algorithm and NSGAII^36^.

For multi-objective big data problems, An Automated differential evolution as a local search that enhances exploration is used for both multi-objective and single-objective problems^37^. Besides, a hybrid of SSA and differential evolution is used to enhance the exploitation of SSA processed for multi-objective big data problems^38^. An adapted indicator method of inverted generational distance processed for variant multi-objective problems with many different shapes of the Pareto front improved the performance of the evolutionary algorithms based on the referenced point method^39^. But the balanced multi-objective optimization algorithm of the referenced points’ method is proposed by Abdel-Basset et al.^40^. Based on the preferences of a decision maker technique, the multi-objective interactive evolutionary algorithms are integrated for many interval problems^41^. Although the goal selection repository is integrated into the multi-objective grasshopper algorithm^42^. The leader selection method to update non-dominated solutions is an integrated multi-objective artificial sheep algorithm^43^.

An alternative repository is integrated into a multi-objective particle swarm optimization algorithm^44^., while handling the full repository by removing solutions from the least populated regions, is integrated in a multi-objective Salp swarm optimization algorithm for solving the engineering applications^5^. But the crowding distance method to enhance diversity of non-dominated solutions, producing different non-dominated solutions for engineering applications, is integrated in the multi-objective sine-cosine optimization algorithm^45^. A hybrid PSO and genetic algorithm is integrated to enhance local search for more distributed non-dominated solutions through less crowded regions^46^. While multiple thresholding levels for image segmentation are integrated for the whale optimization algorithm^47^.

While swarm optimization algorithms can find sufficient solutions for complex, simple, and multi-objective problems. The Multi-Objective Sparrow Search algorithm, which is one of the most recent multi-objective swarm algorithms that has reached sufficient performance for the carbon fiber drawing process that proposed a scouter strategy while the algorithm searched for the optimal set with an accelerating convergence and better diversity^48^. Besides^49^, provided a comprehensive review of the sparrow search algorithm (SSA), detailing its core principles, key variants, including hybrid, chaotic, and multi-objective versions and diverse applications in fields such as machine learning and energy systems, while also outlining prospective research directions.

In the same context, many optimization algorithms are proposed for multi-objective problems with multiple search mechanisms, such as the multi-objective harmony search algorithm (MHS)^50^, multi-objective whale algorithm (MWO)^51^, the multi-objective crow search algorithm, and the multi-objective flower pollination algorithm.

Despite the proliferation of multi-objective metaheuristics, the pursuit of more efficient and robust optimizers remains a central challenge in the field, as dictated by the No-Free-Lunch theorem^26^. Many existing algorithms still struggle with achieving an optimal balance between convergence speed and solution diversity, particularly with problems with complex Pareto front geometries, such as those that are disconnected, concave, or non-uniform. Furthermore, the performance of swarm-based algorithms is heavily dependent on their core search mechanisms; while some excel in exploration, they may lack the focused exploitation needed for precise convergence, and vice versa. This persistent trade-off underscores the need for novel algorithms that incorporate more sophisticated and balanced search strategies from their inception.

Recently, a novel Sinh-Cosh optimizer(SCHO) as a single objective optimization algorithm has been proposed to tune the controller of automatic voltage regulation to be more stable^52^. While SCHO has been proposed for photovoltaic-thermal power systems, it has also^53^. But the arithmetic optimization algorithm has been integrated with SCHO to enhance SCHO’s performance for better prediction of biological activities^54^. SCHO has also been proposed for aircraft pitch control and steam condenser systems in power plants to enhance the performance of their controller^55,56^. Besides, the Sinh-Cosh method has been integrated with the Dung Beetle Optimization algorithm for the global optimization problem^57^.

A Sinh Cosh Optimizer (SCHO)^7^ represents a recent and innovative entry into the meta-heuristic landscape, distinguished by its foundation in the mathematical properties of the hyperbolic sine and cosine functions. Its promising performance in single-objective optimization stems from a sophisticated architectural design that provides several key advantages over more conventional algorithms.

The primary advantage of SCHO lies in its structured and multi-faceted approach to managing the exploration-exploitation balance. Unlike many algorithms that rely on a single, often linear, transition between these phases, SCHO employs two dedicated phases for both exploration and exploitation. This granular structure allows for more specialized and effective search behaviors: the first phases perform broad, coarse-grained searches, while the second phases conduct intensive, fine-grained searches within promising regions. This is a more nuanced strategy than found in algorithms like PSO or GWO, leading to a more robust search process that is less likely to stagnate at local optima. Furthermore, the adaptive switching mechanism, governed by parameter A, ensures a dynamic and timely transition between these phases throughout the optimization run, rather than being dependent on a simple iteration count.

A second critical advantage is the dynamic bounded search strategy. By leveraging not only the global best solution but also the second-best solution, SCHO intelligently contracts the search space around the most promising regions. This mimics a focused hunting strategy, dramatically accelerating convergence by preventing the dispersion of candidate solutions into unproductive areas of the search space. This is a more focused approach compared to the often fixed or linearly decreasing boundaries in other algorithms. Finally, the use of Sinh and Cosh functions within the position update equations is not merely a novelty; these functions, with their exponential nature, facilitate a wider range of movement dynamics. They can generate more aggressive jumps during exploration for better space coverage and more delicate adjustments during exploitation for precise convergence, offering a search dynamic that is distinct from the linear, trigonometric, or purely random movements prevalent in other swarm-based methods.

The combination of these characteristics, granular phase management, intelligent search space bounding, and unique mathematical drivers, equips SCHO with a powerful and balanced search capability. It is this demonstrated potential for rapid convergence coupled with strong resilience to local optima in single-objective problems that provides the compelling motivation to explore its extension into the multi-objective domain.

According to the SCHO’s combined characteristics and its performance at single objective optimization problems. That proves SCHO’s promising performance, in which SCHO’s balance between exploration and exploitation accelerates convergence through a small number of iterations. A bounded search strategy that is responsible for directing candidate solutions toward the global optimum. And the switching between different phases of exploration and exploitation keeps better diversity while accelerating convergence. These characteristics improve the algorithm’s ability to achieve better solutions for complicated problems. So, according to the advantages of SCHO’s last-mentioned characteristics. The decision is taken according to the reasonably clear idea to extend SCHO for multi-objective problems.

SCHO characteristics achieve many benefits as follows:

- The Sinh and Cosh methods are used to direct solutions in the direction of optimal solutions.
- Not only depends on global solutions while updating populations, but it also uses second optimal solutions to bound the search space during updating populations.
- Bounded strategy resizes the bounded search for a smaller range towards an optimal solution. Many phases of exploration and exploitation allow a variety of updated candidate solutions to focus more on the global optimal and avoid existing in local optima.
- Switching strategy between phases of exploration and exploitation achieves a balance during search between exploration and exploitation methods.
- The combination of these strategies enhanced SCHO performance for fast convergence and escaping from local optimum solutions.
- So SCHO is a good chance to find the optimal solution for many complicated applications.

In the same context, expanding SCHO for multi-objective optimization is expected to achieve good solutions for multi-objective complicated problems. However, there are many proposed multi-objective algorithms for many design problem models for engineering, medical, and industrial applications. When working for MOSCHO’s, the main question is what the need is for this algorithm to be processed for real-world applications. The answer to this question is based on the NFL theorem, which indicates there is no meta-heuristic algorithm that can solve all existing applications with sufficient accuracy for these design applications. And many of the design problems still need more research to obtain better solutions for better performance between the many problem’s objectives and the problem’s constraints.

There are many limitations for multi-objective problems, such as multiple objectives that need to be optimized simultaneously. While testing environments vary according to the applications and their constraints. To handle these limitations for multi-objective problems, there is a need for more multi-objective algorithms that have more ability to handle these limitations. So MOSCHO is proposed based on its characteristics to be used for design problems. According to MOSCHO’s characteristics. It has more ability to handle different complex problems.

So, A memorized multi-objective Sinh-Cosh optimization algorithm (MOSCHO) is a distinct approach proposed in this paper. A method of MOSCHO depends on the Pareto dominance of the non-dominated achieved solutions with a list of leaders that are similar to Multi-objective Particle Swarm Optimization (MOPSO)^28^. Besides the last-mentioned method, a memorized optimal solution can be integrated to bound the search objective towards optimal solutions of many mathematical problems or real applications. So, the proposed approach MOSCHO has a combination of using a leader solution beside memorized local solution in updating positions of population, which helps generate solutions close to the global optimum Pareto set (Pareto front). The experiment is performed on several variant mathematical bi-objectives and tri-objectives, well-known multi-objective benchmark problems, and real-world engineering problems. The proposed MOSCHO demonstrates its ability to solve complicated multiple objective problems in the comparison of some well-known multi-objective algorithms.

The key differentiator of MOSCHO is the integration of this powerful search engine with a ‘memorized’ local search technique. While other multi-objective algorithms use a global leader or a repository, MOSCHO uniquely bounds the search for each candidate solution using both the global leader (from the repository) and its own personal best-found position. This dual guidance system allows for a more nuanced and focused convergence towards the Pareto front while the switching mechanism between multiple exploration and exploitation phases maintains population diversity. Therefore, MOSCHO is not merely another multi-objective variant but a distinct approach that leverages a novel, mathematically-grounded search strategy to simultaneously enhance both convergence and coverage.

The main contributions of this article are as follows:

1. Firstly, extending SCHO for multi-objective optimization to propose a new MOSCHO according to their characteristics to maintain better diversity and convergence.
2. The memorized local optimal solutions proposed to bound the search strategy and focus solutions towards the global optimum.
3. Test experiments are processed for unconstrained mathematical benchmark functions ZDT, DTLZ, CEC2009, CEC2020, and both SRN and welded beam as constrained applications. the performance is checked using many metrics, varied for convergence metrics, coverage metrics, and success performance indicators.
4. Eventually, test the proposed method to solve two real-world electrical engineering applications as the optimal power flow and the Optimal Setting of the Droop controller.
5. Compare the MOSCHO’s performance with the state-of-the-art algorithms as MHS, MWO, MALO, MSSA, MOPSO, and MOSCA.

The remainder of this article has sections as follows:

Section 2 presents the conventional Sinh-Cosh optimizer. Section 3 presents the proposed approach of a novel memorized multi-objective Sinh Cosh algorithm for multi-objective optimization. Section 4 presents performance metrics and MOSCHO parameter sensitivity. Section 5 presents the results and their analysis. Section 6 presents the conclusion of the work.

## Sinh-Cosh algorithm

SCHO^7^ It is a meta-heuristic algorithm inspired by Sinh and Cosh mathematical functions, suggested in 2023. SCHO performs as other meta-heuristic algorithms. The meta-heuristic algorithm starts with random positions and updates each value at each iteration towards the global optimal solution. So at the last iteration, the meta-heuristic algorithm suggests its optimal solution. The search mechanism of the meta-heuristic algorithm is to achieve a balance between two main processes, exploration and exploitation, trying to reach the optimal solution during iterations.

However, SCHO acts as a meta-heuristic operation while performing many steps, such as switching between variant phases of exploration and exploitation processes, and applying a bounded strategy. These steps are combined to achieve a balance between exploration and exploitation processes while updating positions and optimizing the search space towards the optimal solutions.

This balance helps solutions escape from local optima. SCHO depends on executing many steps,

Like all Meta-Heuristic algorithms, the SCHO algorithm randomly initializes positions of multiple candidate solutions (N) within the boundary range, between the upper boundary Ub and the lower boundary Lb, where X refers to the position vector of each solution. Each position vector consists of many variables, which refers to the dimension (D) of the position vector as in Eq. (2).

2 $$\:X=rand\left(N,D\right)*\left(Ub-Lb\right)+Lb$$

where rand has a value in the range 0 to 1.

The SCHO algorithm updates positions based on the last position and the last global solution reached at the last iteration. Where the two phases of exploration are represented in Eqs. 3 and 6, where i refers to the number of solutions between populations, j refers to the index of the position variable, and t refers to the current iteration, $\:{X}_{(i,j)}^{t\:}$refers to the current position of the i_th_ solution in the current iteration t, $\:{X}_{(i,j)}^{t+1}$ refers to the update of the position for the next iteration, $\:{X}_{best}^{j}$ refers to the dimension index for the optimal solution obtained from the last iteration, random values r_1_ and r_2_ have values in the range 0 to 1.

3 $$\:{X}_{(i,j)}^{t+1}=\:\left\{\begin{array}{c}{X}_{best}^{j}+\:{r}_{1}\times\:\:{W}_{1}\:\times\:{X}_{(i,j)}^{t}\:\:\:\:\:\:\:{r}_{2}>0.5\\\:{X}_{best}^{j}-\:\:{r}_{1}\times\:\:{W}_{1}\:\times\:{X}_{(i,j)}^{t}\:\:\:\:\:\:\:{r}_{2}<0.5\end{array}\right.$$

4 $$\:{W}_{1}=\:{r}_{3}\times\:{a}_{1}\times\:\left(cosh\:{r}_{4}+u\:\times\:sinh{\:r}_{4}-1\right)$$

5 $$\:{a}_{1}=\:3\:\times\:\left(-1.3\:\times\:\:\frac{t}{\mathrm{m}\mathrm{a}\mathrm{x}\_iteration}+\:m\right)$$.

, $\:\epsilon\:$ refers to a positive number for the second phase exploration, and from the range 0 to 1 $\:\epsilon\:=0.003$is the recommended value from the experiment of this article, as indicated in Sect. 4.3, $\:{W}_{1}an{d\:W}_{2}\:$The weight coefficients of exploration phase 1 and phase 2, respectively, as in Eqs. 4 and 7, random values r_3_,r_4_,r_5,_ and r6 have values in the range 0 to 1. Where m and u are sensitive coefficients to control the accuracy of exploration or exploitation in the first phase. But parameter n controls the accuracy of exploration in the second phase. Tuning of m, u, and n for this experiment indicated in Sect. 4.3.

6 $$\:{X}_{(i,j)}^{t+1}=\:\left\{\begin{array}{c}{X}_{(i,j)}^{t}+\left|\epsilon\:\times\:\:\:{W}_{2}\:\times\:\:\:\:{X}_{best}^{\left(j\right)}\:\:\:\:-{X}_{(i,j)}^{t}\right|\:\:\:\:\:\:\:\:{r}_{5}>0.5\\\:{X}_{(i,j)}^{t}-\left|\epsilon\:\times\:\:\:{W}_{2}\:\times\:\:\:\:{X}_{best}^{\left(j\right)}\:\:\:\:-{X}_{(i,j)}^{t}\right|\:\:\:\:\:\:\:\:{r}_{5}>0.5\end{array}\right.$$

7 $$\:{W}_{2}=\:{r}_{6}\times\:{a}_{2}$$

8 $$\:{a}_{2}=\:2\:\times\:\left(-\:\frac{t}{\mathrm{m}\mathrm{a}\mathrm{x}\_iteration}+\:n\right)$$.

And it also has two exploitation phases, as in Eqs. 9 and 11. Where r_7_, r_8_, r_9_, r_10,_ and r_11_ have values in the range 0 to 1.

9 $$\:{X}_{(i,j)}^{t+1}=\:\left\{\begin{array}{c}{X}_{best}^{j}+\:{r}_{7}\times\:\:{W}_{3}\:\times\:{X}_{(i,j)}^{t}\:\:\:\:\:\:\:{r}_{8}>0.5\\\:{X}_{best}^{j}-\:\:{r}_{7}\times\:\:{W}_{3}\:\times\:{X}_{(i,j)}^{t}\:\:\:\:\:\:\:{r}_{8}<0.5\end{array}\right.$$

10 $$\:{W}_{3}=\:{r}_{9}\times\:{a}_{1}\times\:\left(cosh\:{r}_{10}+u\:\times\:sinh{\:r}_{10}\right)$$

11 $$\:{X}_{(i,j)}^{t+1}=\:{X}_{(i,j)}^{t}+{r}_{11}\times\:\:\frac{\mathrm{sinh}{r}_{12}}{\mathrm{cosh}{r}_{12}}\:\left|{W}_{2}\:\times\:\:\:\:{X}_{best}^{\left(j\right)}\:\:\:\:-{X}_{(i,j)}^{t}\right|\:$$

While two phases of exploration and two phases of exploitation are based on Sinh and Cosh methods formulas at W_1_ and W_3_ for the first phases of either exploration or exploitation.

At each iteration, the SCHO bounded search strategy mimics the last stage of animal hunting through optimization of the search space according to available range. SCHO initializes the search space as in Eq. 12 and updates the potential space as in Eq. 13 within each iteration. So, the updated Ub and Lb are calculated as in Eqs. 14 and 15, within each iteration, to bound the search space.

12 $$\:{BS}_{1}=floor\:\left(\frac{\mathrm{m}\mathrm{a}\mathrm{x}\_iteration}{\beta\:}\right)$$

Where $\:\beta\:$ customizes initialization of the bounded search space.

13 $$\:{BS}_{k+1}={BS}_{k}+\:floor\:\left(\frac{\mathrm{m}\mathrm{a}\mathrm{x}\_iteration\:-\:\:\:\:{BS}_{k}\:}{\alpha\:}\right)$$

Where k is a positive value, the starting value equals 1, $\:\alpha\:$ controls exploration and exploitation accuracy in potential space.

14 $$\:{Ub}_{k}={X}_{best}^{\left(j\right)}+\:\:\left(1-\:\frac{\mathrm{t}\:\:\:}{\mathrm{m}\mathrm{a}\mathrm{x}\_iteration}\right)\times\:\:\left|{X}_{best}^{\left(j\right)}-\:{X}_{second}^{\left(j\right)}\right|$$

15 $$\:{Lb}_{k}={X}_{best}^{\left(j\right)}-\:\:\left(1-\:\frac{\mathrm{t}\:\:\:}{\mathrm{m}\mathrm{a}\mathrm{x}\_iteration}\right)\times\:\:\left|{X}_{best}^{\left(j\right)}-\:{X}_{second}^{\left(j\right)}\right|$$

Where $\:{X}_{second}^{\left(j\right)}$ represent a sub-optimal solution. Also, Eqs. 13 and 14 are used to rearrange the limits of searching boundaries.

Meta-heuristic algorithm explores solutions within early iterations and exploits solutions in the last iterations. But, switching step processing is allowed to allow SCHO exploring besides exploiting within all iterations to avoid local optimal solutions. A is a switch parameter to allow SCHO to focus on exploration and a small amount of exploitation at early iterations, and in contrast, at the last iterations. A represents as in Eq. 16.

16 $$\:A\:=\:\left(p\:-q\:\times\:{\left(\frac{\mathrm{t}\:\:\:}{\mathrm{m}\mathrm{a}\mathrm{x}\_iteration}\right)}^{\left(\frac{\mathrm{cosh}\frac{\mathrm{t}\:\:\:}{\mathrm{m}\mathrm{a}\mathrm{x}\_iteration}}{\mathrm{sinh}\frac{\mathrm{t}\:\:\:}{\mathrm{m}\mathrm{a}\mathrm{x}\_iteration}}\right)}\:\right)\times\:\:\:\:{r}_{13}$$

Where p and q control the balance between exploration and exploitation, tuning of p and q is indicated in Sect. 4.3.

So, A handles the switching step between exploration and exploitation. But, T handles two phases of exploration and two phases of exploitation as in Eq. 17. In early iterations, the algorithm executes the first phase of exploration and exploitation of solutions towards optimal solutions, and the second phase executes deep exploration and exploitation within the potential search space. Also, $\:{BS}_{k}$ bounds search space of the problem and diversifies solutions to the potential space, ct is a setting coefficient working as a switching point in two phases. SCHO repeats these steps until reaching a stop condition, like the maximum number of iterations.

17 $$\:T\:=floor\:\left(\frac{\mathrm{m}\mathrm{a}\mathrm{x}\:\_\mathrm{i}\mathrm{t}\mathrm{e}\mathrm{r}\mathrm{a}\mathrm{t}\mathrm{i}\mathrm{o}\mathrm{n}\:}{ct}\right)$$

As a result of different phases, switching between them and bounded search strategies, fast convergence is achieved due to the switching between variant phases of exploration and exploitation. While SCHO’s ability to escape from local optimum solutions is achieved due to a balance between exploration and exploitation, and a bounded search strategy.

## Memorized multi-objective Sinh-Cosh optimization algorithm (MOSCHO)

MOSCHO is a multi-objective variant of the SCHO algorithm^7^ to solve both constrained and unconstrained multi-objective problems. Many upgrades are required to extend SCHO for multi-objective optimization. The former is an archive of the maximum number of vectors that contains non-dominated solutions for the Pareto optimal set, called the repository. The latter is a leader’s feature. While the search mechanism was upgraded to merge the last-mentioned features with the conventional algorithm strategies for multi-objective problems. The main functions proposed are similar to those used to extend the Particle Swarm Optimization (PSO) algorithm to solve multiple objectives^28^. In which a repository contains the archived Pareto optimal solutions obtained, and the leader selection feature represents another function^28,58^.

While processing a multi-objective algorithm, the archive handles many different cases. At the start of the algorithm, the repository is initialized with the first generation of non-dominated solutions. The repository serves as an archive, updating at each iteration within the algorithm process to preserve non-dominated optimal solutions using a sorting mechanism to form a Pareto optimal set containing new generations of non-dominated solutions. Updating the repository results involves checking the dominance of the existing archive and the resulting Pareto optimal set from the current iteration to obtain an updated version of the repository. So, the repository obtained the supposed non-dominated solutions at the end of the algorithm.

During iterations after updating positions for optimal solutions, new non-dominated solutions were obtained. the repository handles different cases. case 1, while a new generation of non-dominated solutions is less than or equal to the maximum repository size. In that case, the repository obtained all of the new non-dominated solutions. But the other case occurs when the number of new non-dominated solutions exceeds the maximum repository size.

When updating a repository that contains several non-dominating solutions exceeding the allowed repository size, the deletion function is invoked to remove many solutions from the most densely populated area of the repository, considering a predetermined number of each hypercube until reaching the maximum allowed repository size. The deletion function eliminates several repository solutions when the solution is placed outside the hypercube.

Leader solution is a global optimal solution selected from the multiple non-dominated solutions found in the repository. But the leader solution is selected from the least crowded area of the search space. Using a hypercube of lower area population density increases the probability of a leader being chosen for the next iteration. For both selecting the leader function and deletion function, a roulette wheel selection is first used to determine hypercube probability (Pi) as in Eq. 18, where c is an integer constant with a value greater than one. Where selection and deletion functions use different constants. But hypercubes of lower probability are more recommended to select a new leader.

18 $$\:{P}_{i}=\:\frac{C}{{N}_{i}}$$

MOSCHO resulted from the combination of many characteristics that developed SCHO for multi-objective optimization. The SCHO method has a variant of two exploration and two exploitation phases to obtain fast convergence and good performance on many commonly used single objective functions. MOSCHO has many characteristics that require a decision maker method for adding and deleting non-dominated solutions from the repository. Besides the leader selection of an optimal solution method and memorized optimal solutions resulting from the best solution obtained for each candidate solution, which are integrated with the Sinh-Cosh two phases of exploration and two phases of exploitation. In addition to the bounded search strategy and switching strategy between many phases.

However, SCHO used the second minimum or maximum global solution to bound the search space to update the population’s positions. But MOSCHO used the second solution as a memorized best local solution that leads to a good effect on converging the non-dominated set compared to the Pareto front to retain most of the problem properties and constraints. Where the memorized best local solution is the best position reached by each of the populations through the search space. So, for each candidate solution, there is a variable for the best position that achieves the best objective solutions reached till the current iteration.

MOSCHO characteristics formula involves many functions to achieve the target of each optimization algorithm to reach the global solution. MOSCHO has two sets, where the first set initially contains the random populations and the other contains non-dominated solutions of the populations. Where MOSCHO changes the two sets and estimates the global optimum solution eventually for the optimization problem used according to the following steps.

- The MOSCHO populations initialized and MOSCHO search parameters.
- Evaluate the objective functions of each population to determine their fitness.
- Check the dominance of populations to initialize the repository by non-dominated solutions.
- Select the leader solution from the repository according to the last-mentioned leader selection features in this section.
- Update parameter A to switch between exploration and exploitation methods using Eq. 16.
- For each iteration, determine the memorized best local solution to bound search space Ub and Lb and update Ub and Lb using Eqs. 14 and 15, respectively.
- According to the updated value of A, if its value is greater than 3, go to the exploration phases.
- But the value of A is lower than or equal to 3, go to the exploitation phases.
- Then, according to the T value calculated in Eq. 17, if T is greater than or equal to the current iteration value, update the position according to the first phase of exploration or the first phase of exploitation determined from step e and h.
- Evaluate the fitness of populations’ objective functions.
- Check the dominance of newly obtained solutions and the last non-dominated solutions from the repository to obtain new non-dominated solutions for the repository.
- If the number of new non-dominated solutions exceeds the maximum size of the repository, delete some solutions from the repository according to the deletion selection features mentioned earlier in this section until the repository contains only the maximum size of solutions.
- Step d to step l repeated for the number of iterations for the experiment.
- Eventually. The repository obtained the suggested non-dominated solutions as the global optimal solutions.

In the same context, steps of selecting a leader solution, update candidate solutions depending on the leader solution, memorize the local optimal solution, check dominance to update the repository of non-dominated optimal set. In addition to checking if the repository size resulting from updating the maximum repository size for the repository. So, delete some solutions from the repository to keep the number of solutions not exceeding the maximum repository size.

19 $$\:{Ub}_{k}={X}_{best}^{\left(j\right)}+\:\:\left(1-\:\frac{\mathrm{t}\:\:\:}{\mathrm{m}\mathrm{a}\mathrm{x}\_iteration}\right)\times\:\:\left|{X}_{best}^{\left(j\right)}-\:{X}_{memorized}^{\left(j\right)}\right|$$

20 $$\:{Lb}_{k}={X}_{best}^{\left(j\right)}-\:\:\left(1-\:\frac{\mathrm{t}\:\:\:}{\mathrm{m}\mathrm{a}\mathrm{x}\_iteration}\right)\times\:\:\left|{X}_{best}^{\left(j\right)}-\:{X}_{memorized}^{\left(j\right)}\right|$$

**Fig. 2:**
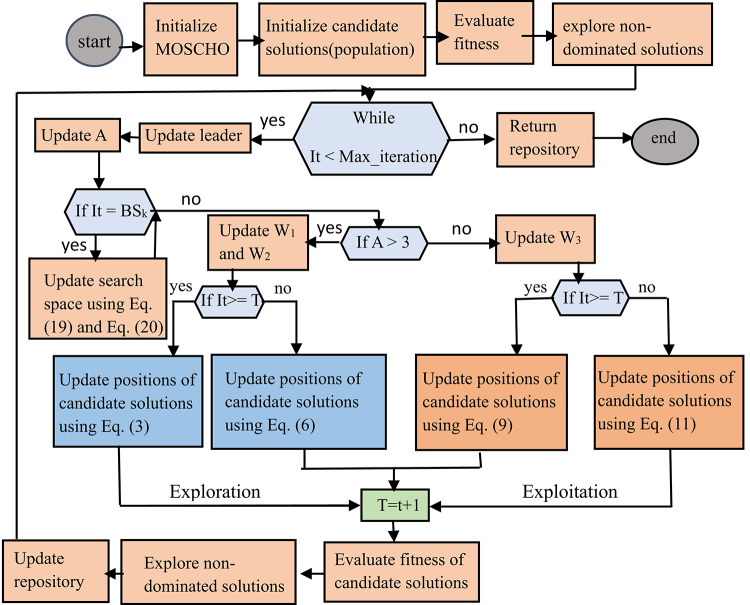
MOSCHO flowchart.

Where $\:{X}_{memorized}^{\left(j\right)}$ represents a memorized local optimal solution, $\:{X}_{best}^{\left(j\right)}$ represents a leader solution, Ub_k_ represents the updated upper boundary, Lb_k_ represents the updated lower boundary, t represents the current iteration, and max_iteration represents the maximum number of iterations to repeat the algorithm’s process. Equations 19 and 20 are used to rearrange the limits of searching boundaries towards the global solution. Bounding the search space fasts convergence and distributes the solutions at the true Pareto front. MOSCHO steps are illustrated in the pseudo code of MOSCHO in Algorithm (1), and the relation between these steps is indicated in both Algorithm (1) and the MOSCHO flow chart, as indicated in Fig. 2.

**Figure:**
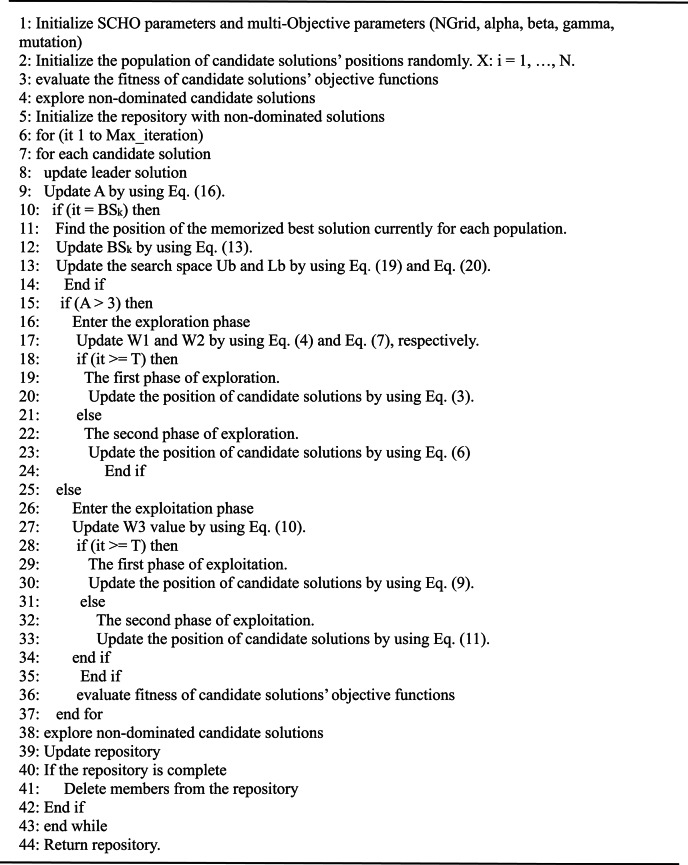
**Algorithm 1 : **Pseudo-code of MOSCHO Algorithm

SCHO computational time complexity is O(N^2^). Although the multi-objective algorithm’s computational time complexity is O(MN^3^), the combination of leader selection and repository archived leads to a computational time complexity of O(MN^2^). Where M represents the number of objectives, but N represents the number of populations.

## Experimental results and discussion

The performance measurements of the proposed algorithm, MOSCHO, which comprises twenty-four experiments, contain nonlinear problems and non-convex complicated problems. MOSCHO algorithm was tested on twenty-two unconstrained multi-objective problems, bi-objective and tri-objective. The experiment includes problems ZDT^59^, DTLZ^60^, CEC 2009^59^ and CEC2020^61^ as test functions. It also includes a constrained multi-objective test problem^62,63^ and a constrained real-world welded beam as an engineering application^64^. They are used to check out MOSCHO availability and efficiency compared to other well-known multi-objective algorithms.

Before MOSCHO’s processing, many of MOSCHO’s parameters required tuning. They let MOSCHO handle multi-objective problems. These parameters are responsible for achieving the best of MOSCHO’s performance for many simple or complicated problems. The tuning process’s results are performed for ZDT problems, and the results are found in Sect. 4.3.

The experiment was conducted on eighteen unconstrained mathematical test problems of bi-objectives and four mathematical test problems of tri-objectives. The details of the mathematical test functions are illustrated as follows. Three ZDT problems, seven CEC2009 problems, nine CEC2020 problems, and three DTLZ problems. Each of the three evaluation problems in ZDT has two goals (objective 1 and objective 2) with thirty variables for ZDT1 and ZDT3, but ZDT4 is processed with only ten variables^59^ where the main details and equations of functions are in Appendix A. While seven CEC 2009 problems have two goals (objective 1 and objective 2) with two variables for each function^65^ where the main details and equations of functions are in Appendix A. But eight of CEC2020 have two goals (objective 1 and objective 2) with two variables for each function, except MMF14, which is processed in three goals (objective 1, objective 2, and objective 3) with three variables^61^ where the main details and equations of functions are in Appendix A. Each of the three evaluation problems in DTLZ has three goals (objective 1, objective 2, and objective 3) with seven variables for DTLZ1 and twelve variables for DTLZ4, but DTLZ6^60^, where the main details and equations of functions are in Appendix A.

They are also tested for two constrained real applications, SRN as a test problem has two goals (objective 1 and objective 2) with two variables^63^ detailed in Appendix B, the welded beam design has two goals (objective 1 and objective 2) with four variables^64^ detailed in Appendix B as a test problem. SRN problem^63^ and welded beam design problem^64^ also has two objectives. These benchmark mathematical functions include the most recent disconnected convex or nonconvex shape functions. They are also used for two electrical real-world applications, the Optimal Power Flow as an electrical design problem that has four goals (objective 1 to objective 4) with thirty-four variables^66^ and the Optimal Setting of Droop controller as an electrical design problem that has three goals (objective 1 to objective 3) with six variables^67^. These two applications are detailed in Appendix C.

### Performance metrics

To evaluate the performance of multi-objective algorithms’ results, many performance metrics are used to analyze convergence and spreading of repository solutions^68,69^.

#### Generational distance (GD)

GD is the total distance between the true Pareto front and the non-dominated optimal set reached from variant approaches. GD is a multi-objective algorithm’s convergence indicator. The algorithm with the smallest value is the best one.

21 $$\:GD=\:\frac{\sqrt{\sum\:_{i=1}^{N}{{d}_{i}}^{2}}}{N}$$

Where N is the number of repository sizes obtained, d_i_ is the Euclidean distance calculated in objective space between the i_th_ obtained Pareto optimal solution and the closest solution from the Pareto front set^70^.

#### Spacing (S)

S determines the distance between the obtained solutions and each other. where the minimum value refers to the best algorithm^71^.

22 $$\:S=\:\sqrt{\frac{1}{N-1}\:\sum\:_{i=1}^{N}{\left(\stackrel{-}{d}-\:{d}_{i}\right)}^{2}}\:$$

Where $\:\stackrel{-}{d}$ represent the average distance of all d_i_ distances.

#### Inverted generational distance (IGD)

IGD is a distance that indicates the quality of the obtained Pareto optimal sets compared to the true Pareto front. The lowest IGD value determines the best algorithm^72^.

23 $$\:IGD=\:\frac{\sqrt{\sum\:_{i=1}^{nt}{{d{\prime\:}}_{i}}^{2}}}{N}$$

Where nt refers to the size of true Pareto optimal solutions, d^’^_i_ is a Euclidean distance calculated in objective space between the i_th_ true Pareto optimal solution and the closest obtained solution from the Pareto optimal set.

#### Hypervolume metric (HV)

HV indicates the algorithm performance of the convergence and the diversity of the obtained Pareto optimal set. HV is a hypercube shape that requires a reference point.

24 $$\:HV=volumne\left(\bigcup\:_{i=1}^{N}{v}_{i}\right)$$

Where the highest HV value refers to the best one^70,73^.

#### Diversity (∆)

The diversity metric analyzes the spreading of the obtained optimal solutions. It represents the average Euclidean distances between the solution and its neighbors of obtained Pareto optimal solutions, considering two extreme boundary solutions of each objective.

25 $$\:\varDelta\:=\frac{{d}_{f}+\:{d}_{l}+\:\sum\:_{1=1}^{N-1}\left|{d}_{i}-\:\stackrel{-}{d}\right|}{{d}_{f}+{d}_{l}+\left(N-1\right)\stackrel{-}{d}}$$

Where d_f_ and dl represent Euclidean distances between the obtained boundary and extreme solutions^29^.

#### Error ratio (ER)

ER metric counts the number of obtained Pareto optimal solutions that existed on the true Pareto front set^74^.

26 $$\:ER=\frac{\sum\:_{i=1}^{n}{e}_{i}}{n}$$

Where e_i_ represents the difference between the obtained Pareto optimal and Pareto front solutions. And n represents the number of solutions for both the Pareto front set and the obtained Pareto optimal set.

#### Success counting (SCC)

SCC metric represents the count of obtained Pareto optimal solutions that are found on the true Pareto front set^72^.

27 $$\:SCC=\sum\:_{i=1}^{n}{s}_{i}\:\mathrm{w}\mathrm{h}\mathrm{e}\mathrm{r}\mathrm{e}\:{s}_{i}=\:\left\{\begin{array}{c}\:1\:\:\:\:\:\:\:\:\:\:\:\:\:\:\:\:\:\:if\:{p}_{i}\in\:TPF\\\:0\:\:\:\:\:\:\:\:\:\:\:\:\:\:\:\:\:\:\:otherwise\end{array}\right.$$

Where pi represents the obtained solution from the Pareto optimal set. TPF represents a true Pareto optimal set. And n represents the number of solutions for both the Pareto front set and the obtained Pareto optimal set.

The non-dominated optimal set obtained from each implemented algorithm is compared to the Pareto front set to analyze convergence and spreading of the Pareto optimal reached set to analyze the algorithm’s performance. Although GD, IGD, and HV metrics quantify convergence, S and ∆ determines coverage of the obtained Pareto optimal solutions by the algorithms. But ER and SCC determine the success performance indicator.

A non-parametric statistical test called Wilcoxon’s Rank-Sum Test (WRT) is used to evaluate relations of many data sets generated by each algorithm in the comparison. For variant algorithms with the same performance metrics, WRT examines the algorithms’ differences using =, +, and - symbols to compare algorithms with each other’s^75^.

In this experiment, A laptop HP of Intel(R) Core (TM) i7-8550U CPU with 1.80 GHz and 12 GB RAM is used to run a program running on Windows 10 of a 64-bit operating system, MATLAB 2021a to perform the operational process.

### MOSCHO’s performance analysis results

MOSCHO’s performance was compared to many relevant algorithms in the literature review, such as NSGAII^29^, Multi-objective Salp Swarm algorithm (MSSA)^5^, Multi-objective Ant Lion optimizer (MALO)^62^, MOPSO^28^, MWO^51^, MHS^50^, and Multi-objective Sine-Cosine Algorithm (MOSCA)^45^. They work at 100 iterations for each algorithm. Where each algorithm initiates with 100 candidate solutions, and the repository obtains only 100 non-dominated solutions. The performance is tested using twenty-two non-constrained mathematical benchmark functions for both eighteen bi-objective and four tri-objective functions. It is also tested in two real-world design problems. Because the constrained problems and non-constrained problems for the experiment have known Pareto front sets called true Pareto optimal sets.

There are many performance metrics used to validate the efficiency of multi-objective algorithms. The used performance metrics vary between convergence metrics, coverage metrics, and the success performance indicator. While the referenced true Pareto optimal sets were used as a reference to the obtained Pareto sets by each algorithm, they were required to evaluate most of the performance metrics used. To obtain the most accurate values for each performance metric at any given algorithm, the operation is repeated fifteen times, and then the average (AV) and standard deviation (SD) parameters of repeated metrics are calculated to validate the performance of each metric. But the difference among the used algorithms is illustrated by Wilcoxon’s Rank-Sum Test (WRT) operating at 5% significance level.

While WRT is a non-parametric statistical test used to evaluate relations of many data sets generated by each algorithm in the comparison. For variant algorithms with the same performance metrics, WRT examines the algorithms’ differences using =, +, and - symbols to compare algorithms with each other. The = symbol refers to no differences in performance between the two used algorithms. But the + symbol indicates a positive difference between algorithms and is used for parameters of minimum value, which refers to the best. The – symbol indicates a negative difference between algorithms and is used for parameters of maximum value, which refers to the best^75^.

#### Analysis results of non-constrained mathematical benchmark functions

**Table 1:** GD statistical results of the mathematical test functions ZDT and CEC2009 functions.

function	GD	MALO	MSSA	MOPSO	MOSCA	MOSCHO
ZDT1	Av	2.3477	2.9755	0.6812	**0.3604**	0.4478
	SD	0.3742	0.2225	**0.1872**	0.2754	0.2677
	WRT	+	+	+	-	
ZDT3	Av	2.4152	3.2808	0.7786	0.6087	**0.1998**
	SD	0.7258	0.3446	0.1951	0.4837	**0.1431**
	WRT	+	+	+	+	
ZDT4	Av	44.8058	78.2432	26.2760	19.2910	**0.2254**
	SD	11.6264	10.5699	12.9262	22.2826	**0.2239**
	WRT	+	+	+	+	
UF1	Av	0.6462	0.7073	0.5690	0**.3970**	0.5632
	SD	**0.1460**	0.2145	0.2243	0.1761	0.1636
	WRT	+	+	-	-	
UF2	Av	0.9281	1.2506	0.5597	**0.4483**	0.5247
	SD	0.3002	0.5900	0.2783	**0.2011**	0.2235
	WRT	+	+	+	-	
UF3	Av	0.5143	**0.3841**	0.5333	0.3921	0.4735
	SD	0.2946	0.1874	0.2187	0**.2140**	0.2550
	WRT	+	-	+	-	
UF4	Av	0.6920	0.6959	0.6758	0**.4318**	0.4948
	SD	0.1387	0.2018	**0.1161**	0.1807	0.1539
	WRT	+	+	+	-	
UF5	Av	0.5676	0.8000	0.6544	0**.5038**	0.5254
	SD	0.2228	0.1623	0**.1014**	0.1852	0.2182
	WRT	+	+	+	-	
UF6	Av	0.7545	0.8181	0.51869	0.6147	**0.4430**
	SD	0.3446	0.1906	0.22278	**0.1724**	0.2565
	WRT	+	+	+	+	
UF7	Av	2.0300	1.9962	1**.6575**	1.9299	2.1417
	SD	0.9357	0.9086	0.7630	0.5430	**0.5090**
	WRT	-	-	-	-	
For WRT	+/-/=	9/1/0	8/2/0	8/2/0	3/7/0	

**Table 2:** GD statistical results of the mathematical test functions: CEC2020 and DTLZ problems.

function	GD	MALO	MSSA	MOPSO	MOSCA	MOSCHO
MMF4	Av	**2.3582**	3.0792	3.0689	3.2100	3.1364
	SD	**0.8739**	1.3233	1.4277	1.0592	1.6264
	WRT	-	-	-	-	
MMF5	Av	0.9702	0.6036	0.6897	**0.5869**	0.6285
	SD	0.1963	0.1381	0.2109	**0.1200**	0.1232
	WRT	+	-	+	-	
MMF7	Av	0.6535	0.4794	0.6486	0.5314	**0.4417**
	SD	0.1905	**0.0778**	0.2550	0.2983	0.1193
	WRT	+	+	+	+	
MMF8	Av	0.8591	**0.5133**	0.6408	0.6738	0.6158
	SD	0.0596	**0.0375**	0.0728	0.2286	0.1724
	WRT	+	-	+	+	
MMF10	Av	0.6496	**0.4846**	0.5892	0.6771	0.5667
	SD	0.1899	0.1741	0.1520	0.1427	**0.0866**
	WRT	+	-	+	+	
MMF11	Av	4.0137	3.8472	**2.8246**	3.2080	3.0642
	SD	0.3135	0.5463	0.8796	**0.1797**	0.5082
	WRT	+	+	-	+	
MMF12	Av	4.3620	1.7303	**1.6394**	1.9511	2.6767
	SD	0.7288	0.6065	0.9274	**0.2509**	1.7326
	WRT	+	-	-	-	
MMF13	Av	0.9466	**0.4009**	0.7248	0.6347	0.7899
	SD	0.1780	0.1518	0.3178	**0.1320**	0.2575
	WRT	+	-	-	-	
MMF14	Av	40.9810	143.7030	45.1695	35.7971	**26.3367**
	SD	12.6016	31.4711	6.0964	9.7742	**7.5025**
	WRT	+	+	+	+	
DTLZ1	Av	0.8734	1.1790	**0.6611**	1.1277	1.1294
	SD	0.4055	0.3666	0.3499	0.2290	**0.1437**
	WRT	-	+	-	+	
DTLZ4	Av	8.2764	8.7314	4.2675	0.2466	**0.1807**
	SD	**0.0349**	0.0631	0.3919	0.1092	0.1301
	WRT	+	+	+	+	
DTLZ6	Av	**1.4075**	1.62302	1.61274	1.5543	1.4516
	SD	0.2420	**0.1981**	0.2256	0.2495	0.43808
	WRT	-	+	+	+	
For WRT	+/-/=	9/3/0	6/6/0	7/5/0	8/4/0	

Tables 1 and 2 show the performance of the generational distance (GD) metric of comparative algorithms for this experiment. GD is a metric evaluating the difference between true Pareto optimal solutions (true PF) and obtained Pareto optimal solutions by each algorithm. For metric GD, MOSCHO has a superior result for eight variant functions, where two functions from ZDT problems, as ZDT3 and ZDT4, two functions from CEC2009 as UF6andUF7, three functions from CEC2020 as MMF7, MMF10, and MMF14, and two functions from DTLZ problems as DTLZ1and DTLZ4. Where obtained solutions exist in the true Pareto front for the mentioned functions.

For ZDT problems, MOSCHO has excellent performance for ZDT3 and ZDT4. Where ZDT1 has better performance using algorithms MOSCA and MPSO. For CEC 2009 problems, MOSCHO has better performance for UF6 and UF7 with MOPSO and MOSCA. Where for UF1 to UF5, MOSCA has better performance, with MOPSO, but MALO and MSSA have better performance for SD of GD and AV of GD, respectively. For CEC 2020 problems, MOSCHO has excellent performance for MMF14 and has better performance for MMF7 and MMF10. But MALO has excellent performance for MMF4, and MSSA has excellent performance for MMF8. For DTLZ problems, MOSCHO has better performance at DTLZ1 and DTLZ4.

While WRT approves that MOSCHO has differences of more than 5% differential, as in Tables 1 and 2. For the GD metric, the best value is the minimum value, where MOSCHO has more than 80% positive differences in eighteen functions. compared to MALO, more than 60% positive differences in fourteen functions) compared to MSSA, more than 65% positive differences in fifteen functions were observed compared to MOPSO. MOSCHO has positive results from the GD metric performance over MALO, MSSA, and MOPSO. But WRT performance of MOSCA is equivalent to MOSCHO, where the number of positive values equals the number of negative values in 11 functions for each one.

MOSCHO has excellent performance for the mentioned functions due to the concurring of MOSCHO performance with functions’ search methods in which memorized solutions drive the search space to be more bounded towards the region of the optimal solutions. While switching mechanisms between exploration and exploitation achieves a balance between variant phases. And variant phases allow MOSCHO to alternate exploration and exploitation methods during iterative search.

So, a Combination of memorized optimal solution, besides global solution, and different phases of exploration and exploitation achieves fast convergence of the obtained Pareto optimal set (obtained PF) compared to the true Pareto front set (true PF).

**Fig. 3:**
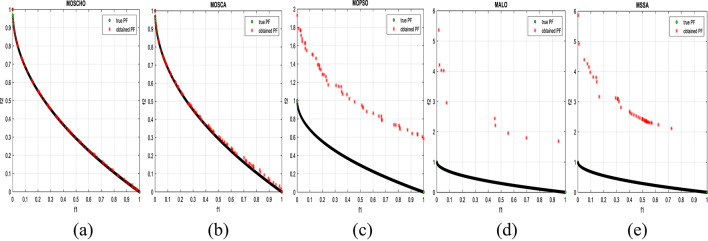
obtained the Pareto optimal set and true Pareto front of the ZDT1 problem. (**a**): MOSCHO, (**b**): MOSCA, (**c**): MOPSO, (**d**): MALO, (**e**): MSSA.

**Fig. 4:**
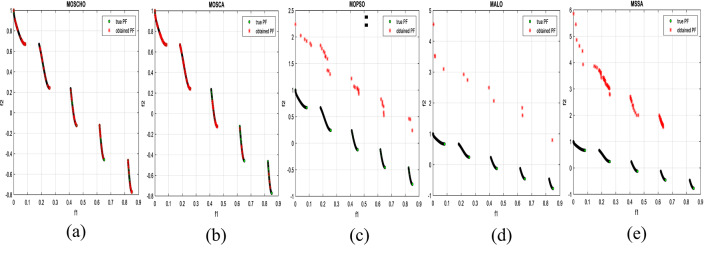
obtained the Pareto optimal set and the true Pareto front of the ZDT3 problem. (**a**): MOSCHO, (**b**): MOSCA, (**c**): MOPSO, (**d**): MALO, (**e**): MSSA.

**Fig. 5:**
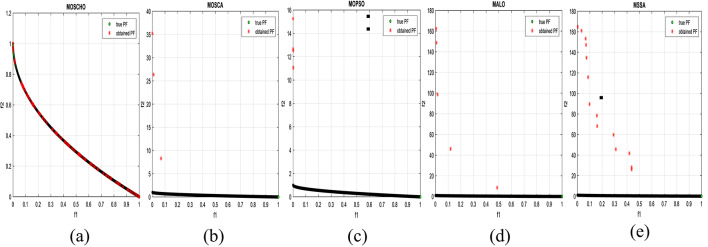
obtained the Pareto optimal set and the true Pareto front of the ZDT4 problem. (**a**): MOSCHO, (**b**): MOSCA, (**c**): MOPSO, (**d**): MALO, (**e**): MSSA.

Figures 3 and 4, and 5 indicate true and obtained Pareto sets for the performance of MOSCHO and other compared algorithms, MOSCA, MPSO, MALO, and MSSA, for ZDT1, ZDT3, and ZDT4 problems. In which MOSCHO has great performance and a great distribution of the obtained set according to the agreement of the multiple objective search space with the MOSCHO search strategies. So, MOSCHO has good performance for both disconnected ranges of several convex functions and good performance for only one convex.

Figure 3 indicates the performance of MOSCHO, in which the repository has non-dominated solutions that achieve fast convergence through the number of iterations used, with the best diversity of the repository compared to the true Pareto front. While MOSCA achieves the nearest performance. But the other compared algorithms achieve poor performance, except MOPSO, which has better diversity among them. This figure proved the MOSCHO’s ability to achieve the best performance for the continuous search space, such as the ZDT1 problem.

Figure 4 indicates MOSCHO’s performance for the separated search spaces. It has a repository of non-dominated solutions with the fastest convergence and the best diversity, in which the obtained Pareto set covers the separated search spaces. Although MOSCA achieves the nearest performance, the others achieve poor performance. This figure proves the MOSCHO’s ability to achieve the best performance for a separated search space, such as the ZDT3 problem.

Figure 5 indicates MOSCHO’s performance for the more complicated problem for a continuous search space, such as the ZDT4. In which the MOSCHO achieves the fastest convergence and the best diversity compared to the other used algorithms. That proves the MOSCHO’s ability for more complicated problems with a continuous search space.

The last three figures indicate that MOSCHO reached the best convergence and best distribution of repository elements compared to true Pareto sets through a hundred iterations. MOSCA has better performance compared to the other compared algorithms. The MOSCHO repository elements are placed and distributed over the whole range of the true Pareto set for ZDT1, ZDT3, and ZDT4 problems. They prove the MOSCHO performance compatibility with these problems. In which MOSCHO’s characteristics direct populations towards the Pareto front set based on the equilibrium between the exploration and exploitation process and bounding search space towards the Pareto front.

However, ZDT3 has more than one convex and not a connected search space. MOSCHO is the best one of the used algorithms for this experiment, which can reach the optimal solutions and apply the greatest distribution of obtained PF on true PF compared to the other used algorithms. MOSCHO focuses the search space in the direction of the optimal solutions early. So MOSCHO has the fastest convergence between the used algorithms.

While ZDT1 has only one convex for its search space. MOSCHO has fast convergence of obtained PF and performs a good distribution of obtained PF to true PF. However, ZDT4 is a more complicated function compared to ZDT1 and ZDT3. ZDT4’s search space has only one convex. MOSCHO has the best performance for ZDT4 for both convergence and diversity of the algorithms.

Because of SCHO characteristics, such as many variant exploration and exploitation phases that enable MOSCHO to reach an optimal solution early. The used switched mechanism has a good alternative way between the exploration and exploitation phases. And memorizing the local optimum solution reduces the search space towards the region containing the optimal solution. While these mechanisms are iterative through the MOSCHO iteration.

So, ZDT1, ZDT3, and ZDT4 have fast convergence and the best diversity of each one. But either ZDT1 or ZDT4 has only one convex. While ZDT3 has disconnected several convex components. The best performance of ZDT1, ZDT3, and ZDT4 proves the MOSCHO’s performance superiority and the agreement between MOSCHO mechanisms and function search spaces.

**Table 3:** IGD statistical results of the mathematical test functions: ZDT and CEC2009 functions.

function	IGD	MALO	MSSA	MOPSO	MOSCA	MOSCHO
ZDT1	Av	1.9083	2.7127	0.5247	**0.2433**	0.2919
	SD	0.4522	0.4292	**0.1560**	0.2135	0.2117
	WRT	+	+	+	-	
ZDT3	Av	1.7493	2.7533	0.6166	0.3795	**0.1217**
	SD	0.5512	0.3865	0.1181	0.3158	**0.0944**
	WRT	+	+	+	+	
ZDT4	Av	35.4166	32.8031	26.2187	16.3533	**0.2769**
	SD	8.2669	15.6605	12.9950	20.0201	**0.2707**
	WRT	+	+	+	+	
UF1	Av	0.3940	0.3854	0.3589	**0.2677**	0.3191
	SD	0.1129	0.1903	0.1879	0.1183	**0.1024**
	WRT	+	+	+	-	
UF2	Av	0.4236	0.5168	**0.3780**	0.4589	0.5401
	SD	**0.1429**	0.1778	0.1722	0.2424	0.2074
	WRT	+	+	+	-	
UF3	Av	0.2842	**0.2194**	0.3361	0.2945	0.3162
	SD	0.1780	**0.1215**	0.1628	0.2396	0.2313
	WRT	-	-	=	-	
UF4	Av	0.4084	0.3689	0.3942	**0.2864**	0.2873
	SD	0.1634	0.1442	**0.1014**	0.1471	0.1079
	WRT	+	+	+	+	
UF5	Av	**0.3483**	0.5269	0.4148	0.3481	0.3603
	SD	0.1716	0.1540	**0.1155**	0.1568	0.1635
	WRT	-	+	+	+	
UF6	Av	0.3373	0.3247	0.3169	0.3654	**0.2833**
	SD	**0.1392**	0.0577	0.1605	0.1461	0.2007
	WRT	+	+	+	+	
UF7	Av	1.5473	1.5487	1.3202	**1.0794**	1.9547
	SD	1.3606	1.4088	1.0766	**0.2732**	1.3710
	WRT	-	-	-	-	
WRT	+/-/=	7/3/0	8/2/0	8/1/1	5/5/1	

**Table 4:** IGD statistical results of the mathematical test functions: CEC2020 and DTLZ functions.

function	IGD	MALO	MSSA	MOPSO	MOSCA	MOSCHO
MMF4	Av	1.6949	**1.6061**	2.0638	2.2782	2.7307
	SD	1.3333	**0.6754**	2.1092	1.2919	2.6135
	WRT	-	-	-	+	
MMF5	Av	0.9427	**0.5171**	0.6032	0.5354	0.6278
	SD	0.1608	0.1399	0.2100	**0.1268**	0.2299
	WRT	+	-	+	-	
MMF7	Av	0.6054	**0.3060**	0.4680	0.3839	0.6078
	SD	0.1924	**0.0361**	0.1884	0.1369	0.2086
	WRT	-	-	-	-	
MMF8	Av	0.8933	0.6130	**0.6040**	0.7388	0.7666
	SD	0.0855	**0.0390**	0.0599	0.1642	0.1228
	WRT	+	-	-	-	
MMF10	Av	0.3860	**0.2939**	0.4092	0.5159	0.3918
	SD	0.1000	**0.0998**	0.1139	0.1267	0.1181
	WRT	+	-	+	+	
MMF11	Av	3.7494	3.6564	**2.6970**	3.1560	2.9018
	SD	0.5309	0.5920	0.7038	**0.2350**	0.4189
	WRT	+	+	-	+	
MMF12	Av	4.0519	1.7278	**1.5345**	1.8666	2.2797
	SD	0.8785	0.4986	0.7224	**0.2493**	1.0885
	WRT	+	-	-	-	
MMF13	Av	1.0016	0.7548	0.7430	0.7256	**0.6693**
	SD	0.1730	0.0953	0.1898	**0.0881**	0.1622
	WRT	+	+	+	+	
MMF14	Av	27.9387	36.4662	32.5733	23.3631	**23.1639**
	SD	16.3189	53.6050	14.1528	16.9351	**12.9485**
	WRT	+	+	+	=	
DTLZ1	Av	0.9395	0.9619	**0.6182**	0.9897	1.0196
	SD	**0.1200**	0.0845	0.2073	0.2100	0.1408
	WRT	-	-	-	-	
DTLZ4	Av	8.0395	8.5773	3.7831	0.2264	**0.1768**
	SD	0.2534	0.1030	0.6426	**0.0894**	0.1199
	WRT	+	+	+	+	
DTLZ6	Av	1.1779	1.1168	1.0816	1.1453	**1.0108**
	SD	0.2624	**0.0753**	0.1528	0.2958	0.3546
	WRT	+	+	+	+	
WRT	+/-/=	9/3/0	5/7/0	6/6/0	6/5/1	

Tables 3 and 4 show the statistical results of the inverted generational distance (IGD) metric for all benchmark functions. For metric IGD, MOSCHO has a better performance of eight variant functions, as two functions ZDT3and ZDT4 from ZDT problems, two functions of CEC2009 as UF1 and UF6, two functions for CEC2020 as MMF13 and MMF14, and two functions of DTLZ problems as DTLZ4 and DTLZ6.

MOSCHO has excellent performance for ZDT3 and ZDT4. However, MOPSO and MOSCA have better performance for ZDT1. MOSCHO has better performance for UF1 and UF6. But other tested algorithms are distributed to obtain better performance for other CEC2009 functions. MOSCHO has excellent performance for MMF14 and better performance for MMF13. MSSA has excellent performance for MMF4 and MMF7. For DTLZ problems, MOSCHO has better performance for DTLZ4 and DTLZ6, but MALO has better performance for DTLZ1.

While WRT approves that MOSCHO has differences of more than 5% differential, as in Tables 3 and 4. For the IGD metric, the best value is the minimum value, where MOSCHO has more than 70% positive differences in sixteen functions. compared to MALO, up to 60% positive differences in thirteen functions) compared to MSSA, more than 60% positive differences in fourteen functions compared to MOPSO, and more than 50% positive differences in 11 functions compared to MOPSO, and there are no differences for function UF3 between both MOSCHO and MOPSO. Because MOSCHO has positive results from the IGD metric performance over MALO, MSSA, and MOPSO. But WRT performance of MOSCA is equivalent to MOSCHO, where the number of positive values equals the number of negative values in 11 functions for each one, and there are no differences for function MMF14 between both MOSCHO and MOSCA.

Due to the good characteristics of MOSCHO, the memorized local search bounded the search space to converge solutions towards the global optimal region. Bounded search and leader optimal solutions selected enhance the convergence of the obtained PF. And switching between variant phases of exploration and exploitation further enhances convergence MOSCHO. So, Tables 3 and 4 confirm the good convergence of the MOSCHO algorithm. And WRT approves MOSCHO as superior in its convergence performance.

**Fig. 6:**
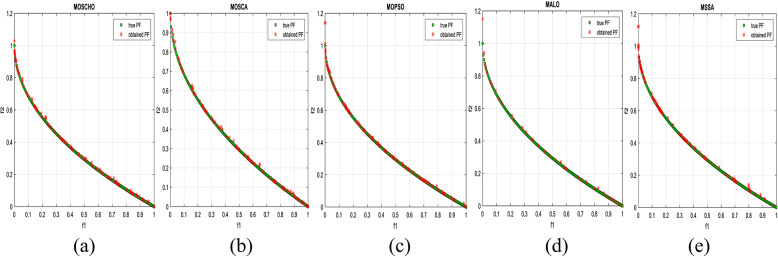
Obtained Pareto optimal set and true Pareto front of UF1 problem. (**a**): MOSCHO, (**b**): MOSCA, (**c**): MOPSO, (**d**): MALO, (**e**): MSSA.

**Fig. 7:**
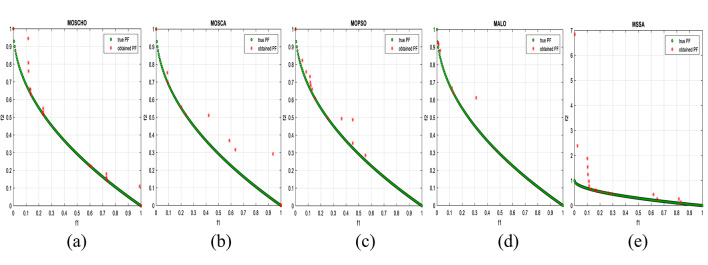
obtained the Pareto optimal set and the true Pareto front of UF2 problem. (**a**): MOSCHO, (**b**): MOSCA, (**c**): MOPSO, (**d**): MALO, (**e**): MSSA.

**Fig. 8:**
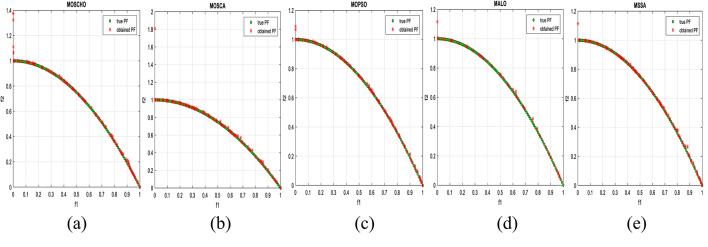
obtained Pareto optimal set and true Pareto front of UF3 problem. (**a**): MOSCHO, (**b**): MOSCA, (**c**): MOPSO, (**d**): MALO, (**e**): MSSA.

**Fig. 9:**
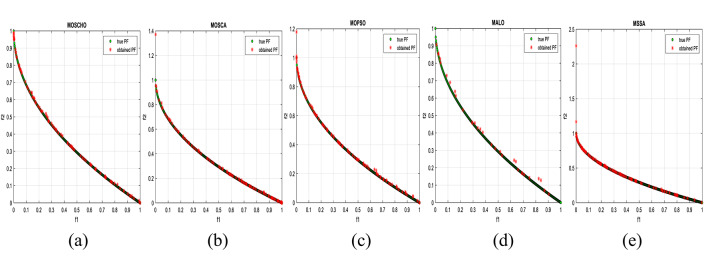
Obtained Pareto optimal set and true Pareto front of UF4 problem. (**a**): MOSCHO, (**b**): MOSCA, (**c**): MOPSO, (**d**): MALO, (**e**): MSSA.

**Fig. 10:**
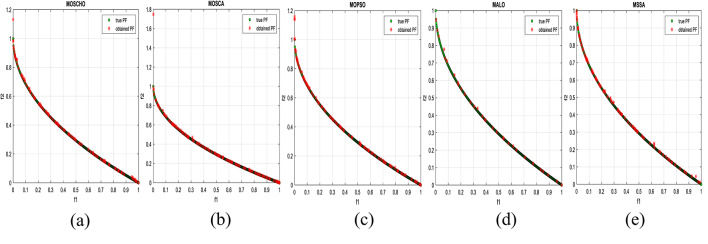
Obtained Pareto optimal set and the true Pareto front of UF5 problem. (**a**): MOSCHO, (**b**): MOSCA, (**c**): MOPSO, (**d**): MALO, (**e**): MSSA.

**Fig. 11:**
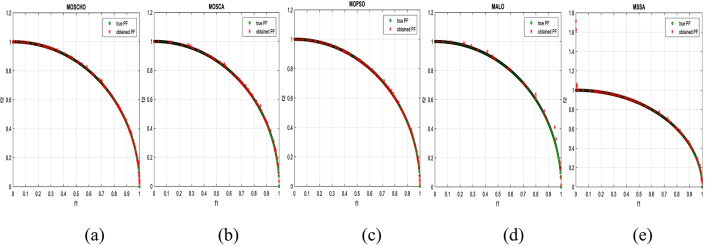
Obtained Pareto optimal set and true Pareto front of UF6 problem. (**a**): MOSCHO, (**b**): MOSCA, (**c**): MOPSO, (**d**): MALO, (**e**): MSSA.

**Fig. 12:**
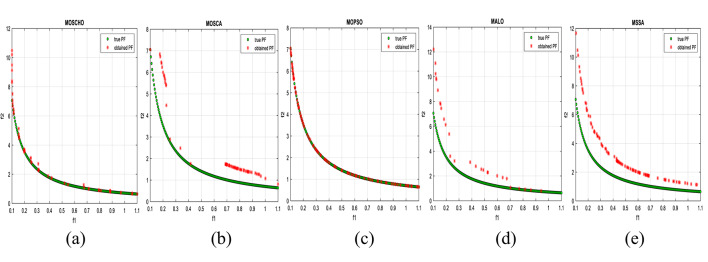
Obtained Pareto optimal set and true Pareto front of UF7 problem. (**a**): MOSCHO, (**b**): MOSCA, (**c**): MOPSO, (**d**): MALO, (**e**): MSSA.

Figures 6, 7, 8, 9, 10, 11 and 12 indicate true and obtained Pareto sets (obtained PF) for the performance of MOSCHO and other compared algorithms, MOSCA, MPSO, MALO, and MSSA. In which MOSCHO has superior performance expressed in a great convergence and great distribution of the obtained set compared to the true Pareto set for all CEC 2009 functions except UF2 and UF7 due to their complicated objective space non-uniform shape that contains many convex/concave components.

MOSCHO has good performance for UF1 to UF6 except UF2. However, they have more than one convex except UF3, and UF6 has more than one concave. And they have regular search sets achieved by MOSCHO as a result of bounded search based on the memorized local solution and a good balance between exploration and exploitation phases.

Figures illustrate fast convergence and good distribution of the obtained PF compared to the true PF. This convergence performance results from bounded search reached from memorized local search solutions and from the variation of balanced distribution of exploration and exploitation phases. These mechanisms also enhance the convergence performance.

But MOSCHO characteristics are not enough to obtain fast convergence and good diversity due to the use of a little inappropriate search method followed by MOSCHO to handle many irregular search sets or the complexity of both UF2 and UF7. UF2 has the most complex irregular search set. in the same context, the other used algorithms perform poorly in terms of convergence and distribution for UF2. But for UF7, MOPSO has the fastest convergence and good distribution, unlike MOSCHO, which has good convergence and lower distribution than MOPSO. That is because MOPSO updates populations based on a combination of global and local optimal solutions.

**Table 5:** Statistical results S of the mathematical test functions: ZDT and CEC2009 functions.

function	S	MALO	MSSA	MOPSO	MOSCA	MOSCHO
ZDT1	Av	0.3741	0.3704	0.3031	**0.2394**	0.2561
	SD	0.1376	0.0901	**0.0472**	0.0474	0.0510
	WRT	+	+	-	-	
ZDT3	Av	0.4445	0.4105	0.3137	**0.2940**	0.3655
	SD	0.1210	0.1022	0.0659	0.0619	**0.0471**
	WRT	+	+	+	-	
ZDT4	Av	7.6630	20.4020	NaN	NaN	**0.2595**
	SD	4.9199	7.6839	NaN	NaN	**0.0504**
	WRT	+	+	+	-	
UF1	Av	**0.2205**	0.2208	0.2515	0.2244	0.2247
	SD	0.0612	0.0563	0.0598	**0.0533**	0.0584
	WRT	+	+	+	+	
UF2	Av	0.5228	0.8825	**0.1771**	0.2826	0.2932
	SD	0.3086	0.6244	**0.0712**	0.1348	0.0873
	WRT	+	+	+	-	
UF3	Av	0.2391	**0.2329**	0.2474	0.2426	0.2571
	SD	0.0550	0.0546	0.0519	0.0536	**0.0482**
	WRT	+	-	-	-	
UF4	Av	0.2276	0.2130	0.2333	0.2360	**0.2024**
	SD	0.0606	0.0517	0.0588	0.0519	**0.0375**
	WRT	+	+	+	+	
UF5	Av	0.2323	0.2239	0.2254	0.2349	**0.2167**
	SD	0.0397	**0.0351**	0.0539	0.0584	0.0405
	WRT	-	+	+	+	
UF6	Av	0.6419	0.5433	0.2204	**0.2058**	0.2478
	SD	0.5317	0.5903	0.0510	**0.0484**	0.0543
	WRT	-	-	-	-	
UF7	Av	1.5296	0.9848	0.9993	**0.8371**	1.0514
	SD	0.3986	0.2358	**0.1741**	0.2216	0.44902
	WRT	+	+	-	-	
WRT	+/-/=	8/2/0	8/2/0	6/4/1	3/7/0	

**Table 6:** Statistical results S of the mathematical test functions: CEC2020 and DTLZ functions.

function	S	MALO	MSSA	MOPSO	MOSCA	MOSCHO
MMF4	Av	1.5614	**1.4863**	1.6872	1.5542	1.7310
	SD	0.4015	**0.2118**	0.4316	0.2512	0.3432
	WRT	-	-	+	-	
MMF5	Av	0.2757	NaN	0.2135	**0.2026**	0.2821
	SD	0.1147	NaN	0.1356	**0.0714**	0.0768
	WRT	+	-	+	-	
MMF7	Av	0.2562	**0.1195**	0.2229	0.1932	0.2886
	SD	0.0632	**0.0284**	0.04648	0.0450	0.0540
	WRT	-	-	-	-	
MMF8	Av	0.1093	0.0906	**0.0403**	0.3375	0.2828
	SD	0.0518	**0.0129**	0.0329	0.0865	0.0427
	WRT	-	-	-	+	
MMF10	Av	0.2379	0.2234	0.2180	0.2715	**0.2013**
	SD	0.0607	0.0423	0.0412	0.0886	**0.0391**
	WRT	+	+	-	+	
MMF11	Av	0.5385	0.2612	**0.1996**	0.2813	0.3623
	SD	0.2723	0.2117	0.2165	**0.1931**	0.2419
	WRT	+	-	-	-	
MMF12	Av	0.8204	NaN	0.1662	**0.1309**	0.3328
	SD	0.3829	NaN	0.3077	**0.2041**	0.2833
	WRT	+	-	+	-	
MMF13	Av	0.2120	NaN	0.2520	**0.1883**	0.3146
	SD	0.0889	NaN	0.1557	0.1309	**0.0790**
	WRT	-	-	=	-	
MMF14	Av	16.1471	45.36906	12.3172	9.0070	**7.1365**
	SD	6.93067	13.0227	3.3848	3.0871	**2.4430**
	WRT	+	+	+	+	
DTLZ1	Av	0.1330	**0.1320**	0.2254	0.3968	0.3874
	SD	0.0876	0.0472	**0.0285**	0.0673	0.0642
	WRT	+	+	+	+	
DTLZ4	Av	1.2946	0.4573	1.0158	0.1916	**0.1767**
	SD	0.2533	0.1689	0.1752	**0.0370**	0.0443
	WRT	+	+	+	+	
DTLZ6	Av	0.2955	**0.1586**	0.2777	0.3588	0.3450
	SD	0.0461	0.0404	**0.0339**	0.0445	0.0466
	WRT	+	-	+	+	
WRT	+/-/=	8/4/0	3/9/0	7/4/1	6/6/0	

Tables 5 and 6 illustrate the statistical results of the spacing performance metric (S) of all used mathematical benchmark functions. From metric spacing’s results, MOSCHO has a better performance of nine variant functions, as two functions, ZDT3 and ZDT4 of ZDT problems, three functions UF3, UF4, and UF5 of CEC2009 functions, three functions MMF10, MMF13, and MMF14 from CEC2020, but only one function DTLZ4 from DTLZ problems, as in Tables 5 and 6.

MOSCHO has excellent performance for ZDT4and better performance for ZDT3. Where MALO, MOPSO, and MOSCA have better performance for ZDT1 to ZDT3. For CEC 2009, MOSCHO has excellent performance for UF4 and better performance for UF3 and UF5, but MOPSO and MOSCA have excellent performance for UF2 and UF6, respectively. For CEC 2020, MOSCHO has superior performance at MMF10 and MMF14. MOSCHO has better performance for MMF13. MOSCA has excellent performance for MMF5 and MMF13. For DTLZ, MOSCHO has better performance for DTLZ4 and DTLZ6, where all tested algorithms are distributed for better performance of DTLZ problems.

While WRT approves that MOSCHO has differences of more than 5% differential, as in Tables 5 and 6. For the spacing metric, the best value is the minimum value, where MOSCHO has more than 70% positive differences in sixteen functions. compared to MALO, more than 50% positive differences in twelve functions compared to MSSA, up to 60% positive differences in thirteen functions compared to MOPSO, and more than 40% positive differences in eleven functions compared to MOPSO, and there are no differences for function MMF13 between both MOSCHO and MOPSO. MOSCHO has positive results from the spacing metric performance over MALO, MSSA, and MOPSO.

As a result of a balance between exploration phases and exploitation phases and bounded search to resize the search space based on both the global optimal solution and memorized global solutions, MOSCHO has a great distribution of obtained Pareto optimal sets, which refers to good coverage of the algorithm.

**Fig. 13:**
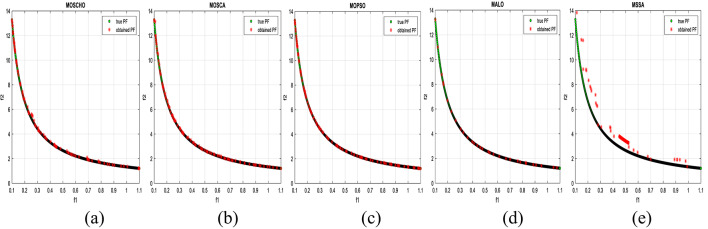
Obtained Pareto optimal set and true Pareto front of MMF4 problem. (**a**): MOSCHO, (**b**): MOSCA, (**c**): MOPSO, (**d**): MALO, (**e**): MSSA.

**Fig. 14:**
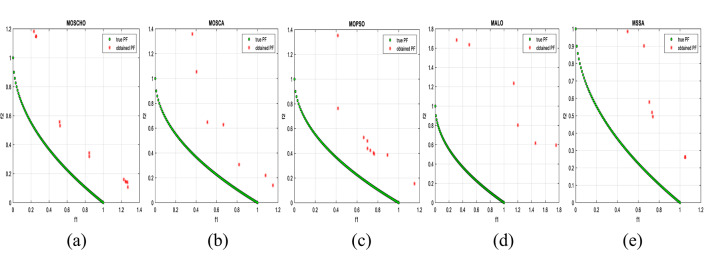
Obtained Pareto optimal set and true Pareto front of MMF5 problem. (**a**): MOSCHO, (**b**): MOSCA, (**c**): MOPSO, (**d**): MALO, (**e**): MSSA.

**Fig. 15:**
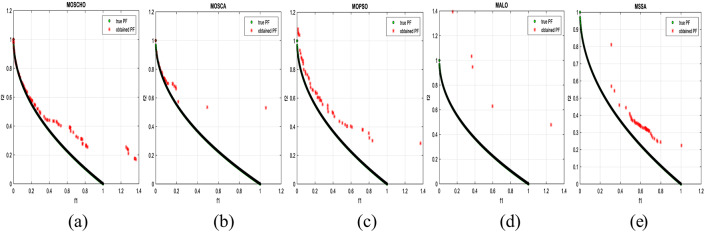
Obtained Pareto optimal set and true Pareto front of MMF7 problem. (**a**): MOSCHO, (**b**): MOSCA, (**c**): MOPSO, (**d**): MALO, (**e**): MSSA.

**Fig. 16:**
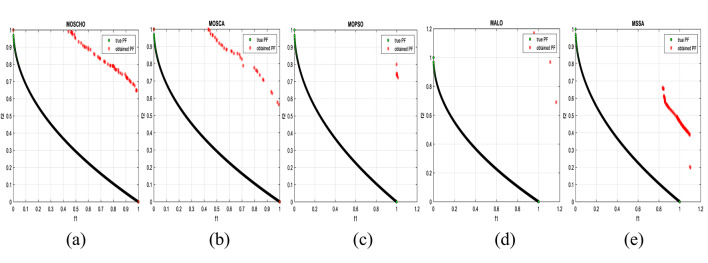
Obtained Pareto optimal set and true Pareto front of MMF8 problem. (**a**): MOSCHO, (**b**): MOSCA, (**c**): MOPSO, (**d**): MALO, (**e**): MSSA.

**Fig. 17:**
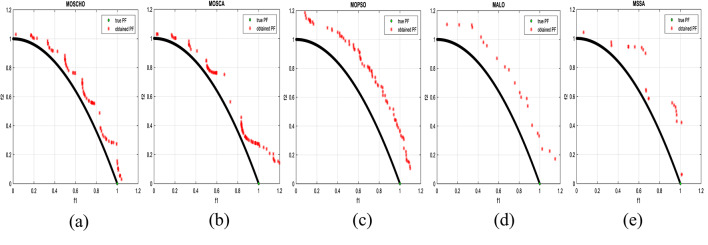
Obtained Pareto optimal set and true Pareto front of MMF10 problem. (**a**): MOSCHO, (**b**): MOSCA, (**c**): MOPSO, (**d**): MALO, (**e**): MSSA.

**Fig. 18:**
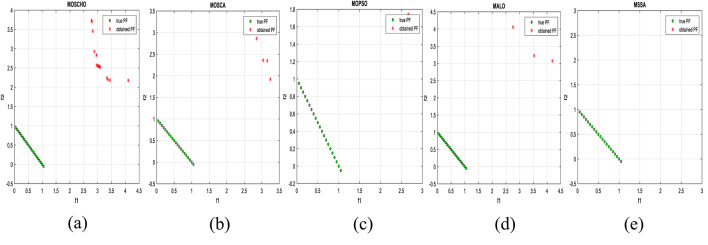
Obtained Pareto optimal set and true Pareto front of MMF11 problem. (**a**): MOSCHO, (**b**): MOSCA, (**c**): MOPSO, (**d**): MALO, (**e**): MSSA.

**Fig. 19:**
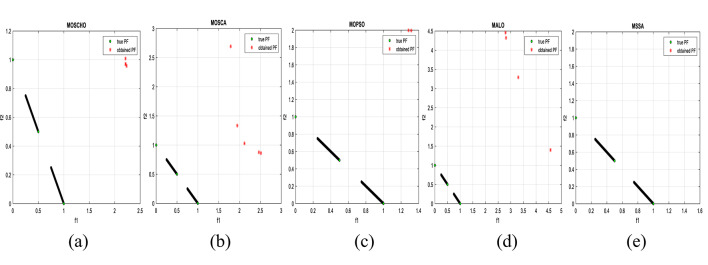
Obtained Pareto optimal set and true Pareto front of MMF12 problem. (**a**): MOSCHO, (**b**): MOSCA, (**c**): MOPSO, (**d**): MALO, (**e**): MSSA.

**Fig. 20:**
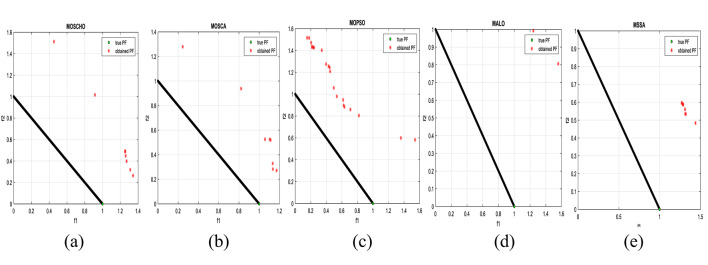
Obtained Pareto optimal set and true Pareto front of MMF13 problem. (**a**): MOSCHO, (**b**): MOSCA, (**c**): MOPSO, (**d**): MALO, (**e**): MSSA.

**Fig. 21:**
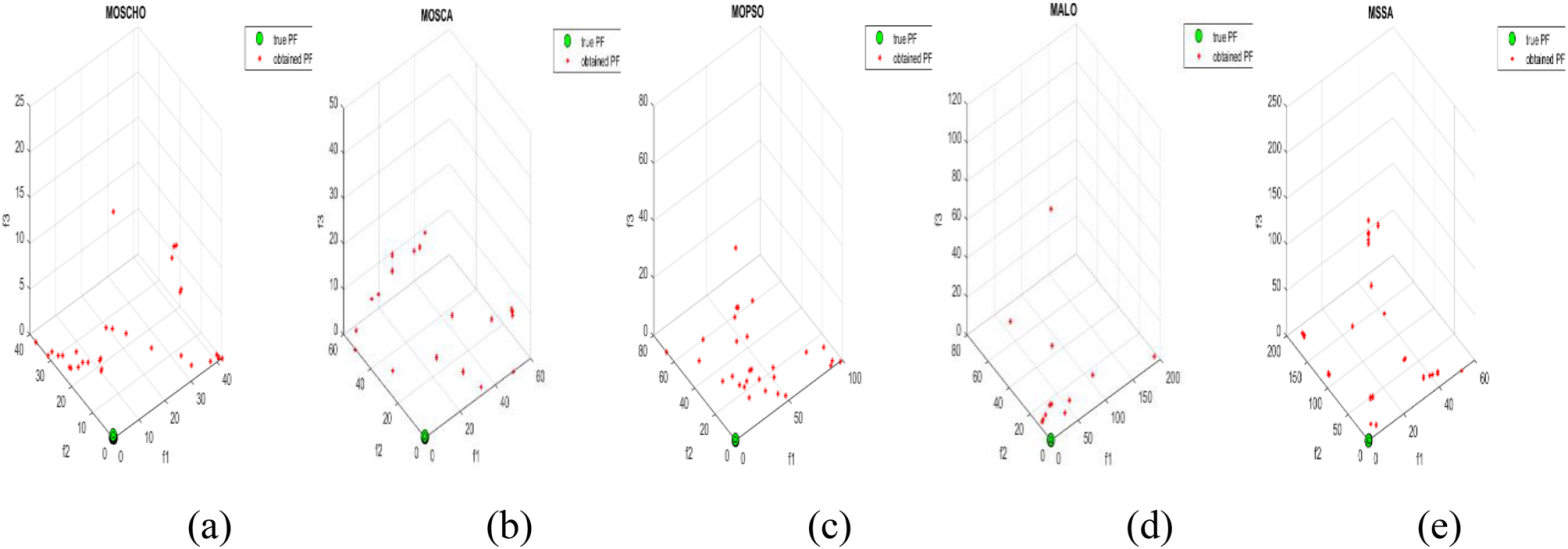
Obtained Pareto optimal set and true Pareto front of MMF14 problem. (**a**): MOSCHO, (**b**): MOSCA, (**c**): MOPSO, (**d**): MALO, (**e**): MSSA.

Figures 13, 14, 15, 16, 17, 18, 19, 20 and 21 indicate true and obtained Pareto sets for the performance of MOSCHO and other compared algorithms, MOSCA, MPSO, MALO, and MSSA. In which MOSCHO has a good performance of the obtained set compared to the true Pareto set. The good convergence and good distribution resulted from MOSCHO characteristics while trying to find non-dominated solutions. But due to the complicated objective search space of MMF5, MMF12, and MMF14, MOSCHO achieves poor convergence and good distribution.

MMF4 has a uniform search space of one convex, and MOSCHO has the best convergence and coverage performance. While MMF5 has an irregular search space of two levels, MOSCHO achieves the best performance of both convergence and coverage compared to other algorithms used. MMF7 and MMF8 have more than one convex and irregular objective space, but MOSCHO achieves applicable convergence and good distribution compared to the other algorithms.

But MMF10 has one concave of a two-level objective space, and MOSCHO had good convergence and good distribution, while MOSCHO handles MMF10 as it has a discrete multi-convex objective space. MMF11 and MMF13 have a linear and complicated objective space. But MOSCHO achieves the best performance.

MMF12 has a multi-convex discrete objective space. All of the used algorithms have poor performance for this function. MMF14 is a three-dimensional convex shape. MOSCHO achieves the best performance compared to other used algorithms for this experiment.

However, MOSCHO has good performance for MMF4 and MMF10 due to its continuous objective space of one convex and applicable performance for MMF5, MMF7, MMF8, and MMF13 due to disconnected objective search, followed by the problems in a complicated objective space. While MOSCHO has good distribution due to and poor convergence for linear discrete objective space problems, such as MMF12.

For CEC2020, MOSCHO achieves the best obtained performance compared to the other used algorithms due to the integrated strategies of MOSCHO that reach these results. But CEC2020 requires more complicated algorithms to handle complicated CEC2020 functions.

**Table 7:** Statistical results HV of the mathematical test functions: ZDT and CEC2009 functions.

function	HV	MALO	MSSA	MOPSO	MOSCA	MOSCHO
ZDT1	Av	0.7376	0.6655	0.9358	0.9996	**0.9998**
	SD	**0.0251**	0.0157	0.0185	0.0005	4.8795e-05
	WRT	-	-	-	-	
ZDT3	Av	0.7122	0.6544	0.8912	**0.9970**	**0.9970**
	SD	0.0178	**0.0285**	0.0271	0.0050	0.0093
	WRT	-	-	-	-	
ZDT4	Av	0.8341	0.8751	0.8798	0.9263	**1**
	SD	0.0396	0.0187	0.0516	**0.0927**	0
	WRT	+	+	-	+	
UF1	Av	0.9973	0.9972	**0.9998**	0.9997	**0.9998**
	SD	0.0014	**0.0026**	3.5186e-05	0.0001	4.1403e-05
	WRT	-	-	-	-	
UF2	Av	0.9831	0.9858	0.9884	0.9994	**0.9995**
	SD	0.0078	0.0068	**0.0108**	0.0001	0.0001
	WRT	-	-	+	-	
UF3	Av	0.9873	0.9930	**1.0004**	**1.0004**	1.0003
	SD	0.0062	**0.0073**	0.0002	0.0001	0.0002
	WRT	-	-	-	-	
UF4	Av	0.9960	0.9985	0.99983	0.9996	**0.99984**
	SD	**0.0020**	0.0016	0.0001	0.0001	5.0709e-05
	WRT	-	-	-	-	
UF5	Av	0.9894	0.9921	**0.9972**	0.9971	0.9971
	SD	0.0032	**0.0041**	0.0002	0.0004	0.0002
	WRT	-	-	-	-	
UF6	Av	0.9963	0.9988	0.9991	0.9987	**0.9994**
	SD	**0.0022**	0.0009	0.0006	0.0011	0.0005
	WRT	-	-	+	-	
UF7	Av	0.9897	0.9746	**0.9999**	0.9883	0.9993
	SD	0.0082	0.0062	3.5186e-05	**0.0089**	0.0002
	WRT	-	-	+	-	
For WRT	+/-/=	1/9/0	1/9/0	3/7/0	1/9/0	

**Table 8:** Statistical results HV of the mathematical test functions: CEC2020 functions.

function	HV	MALO	MSSA	MOPSO	MOSCA	MOSCHO
MMF4	Av	0.9971	0.9779	0.9998	0.9997	**0.9990**
	SD	0.0016	**0.0050**	5.6061e-05	0.0001	0.0007
	WRT	-	-	+	+	
MMF5	Av	0.7183	0.7444	0.8011	0.8754	**0.8826**
	SD	0.0357	**0.0449**	0.0417	0.0318	0.0356
	WRT	-	-	+	-	
MMF7	Av	0.7505	0.8435	0.9058	0.9294	**0.9442**
	SD	0.0280	**0.0465**	0.0135	0.0094	0.0093
	WRT	-	-	-	-	
MMF8	Av	0.6936	0.8112	0.7535	0.9755	**0.9767**
	SD	**0.0170**	0.0128	0.0161	0.0042	0.0004
	WRT	-	-	+	-	
MMF10	Av	0.7663	0.7807	0.7876	0.8595	**0.9158**
	SD	0.0064	0.0503	0.0073	**0.0086**	0.0027
	WRT	-	-	+	-	
MMF11	Av	0.4962	0.4813	0.5875	0.5737	**0.5921**
	SD	0.0470	0.0459	**0.0604**	0.0304	0.0477
	WRT	-	-	+	-	
MMF12	Av	0.6955	0.7987	**0.8319**	0.8256	0.8096
	SD	0.0390	0.0439	0.0428	0.0276	**0.0897**
	WRT	-	-	+	+	
MMF13	Av	0.6317	0.6750	0.7754	0.8422	**0.8791**
	SD	0.0290	0.0395	**0.0785**	0.0578	0.0447
	WRT	-	-	+	-	
For WRT	+/-/=	0/8/0	0/8/0	7/1/0	2/6/0	

Tables 7 and 8 illustrate the statistical results of the hyper volume metric (HV) according to a reference point of each bi-objective problem. From metric hyper-volume’s results, MOSCHO has a better performance of fifteen variant functions of eighteen functions for two objectives, as three functions ZDT problems, seven functions of CEC2009 functions, and eight functions of CEC2020, as shown in Tables 7 and 8.

However, MOSCHO has better performance in ZDT1, ZDT3, and ZDT4 of the three ZDT problems. MOSCHO has UF1, UF2, UF4 and UF6 of seven CEC2009 functions. But MOSCHO has better performance of eight functions from MMF4, MMF5, MMF7, MMF8, MMF10, MMF11, MMF12, and MMF13.

While WRT approves that MOSCHO has differences of more than 5% differential, as in Tables 7 and 8. For the hyper-volume metric, the best value is the maximum value. where MOSCHO has more than 90% negative differences in 17 functions) compared to MALO, more than 90% negative differences in seventeen functions compared to MSSA, more than 65% negative differences in twelve functions compared to MOPSO, and more than 80% negative differences in 15 functions compared to MOSCO. MOSCHO has negative results from the hyper-volume metric performance over MALO, MSSA, MOPSO, and MOSCA.

From Tables 7 and 8, the hyper-volume metric of MOSCHO has a superior performance for functions except for fifteen benchmark problems. MOSCHO has a superior performance for the five mentioned ZDT functions, for seven CEC2009 processed problems except UF2 and UF7, and for ten bi-objective problems of CEC2020 processed. So, the MOSCHO achieves Great coverage results due to the integrated characteristics of memorized best solutions to bound the search space and balance the exploration phases and exploitation phases. These integrated characteristics make it easy to reach optimal solutions with the best diversity of obtained Pareto sets.

**Fig. 22:**
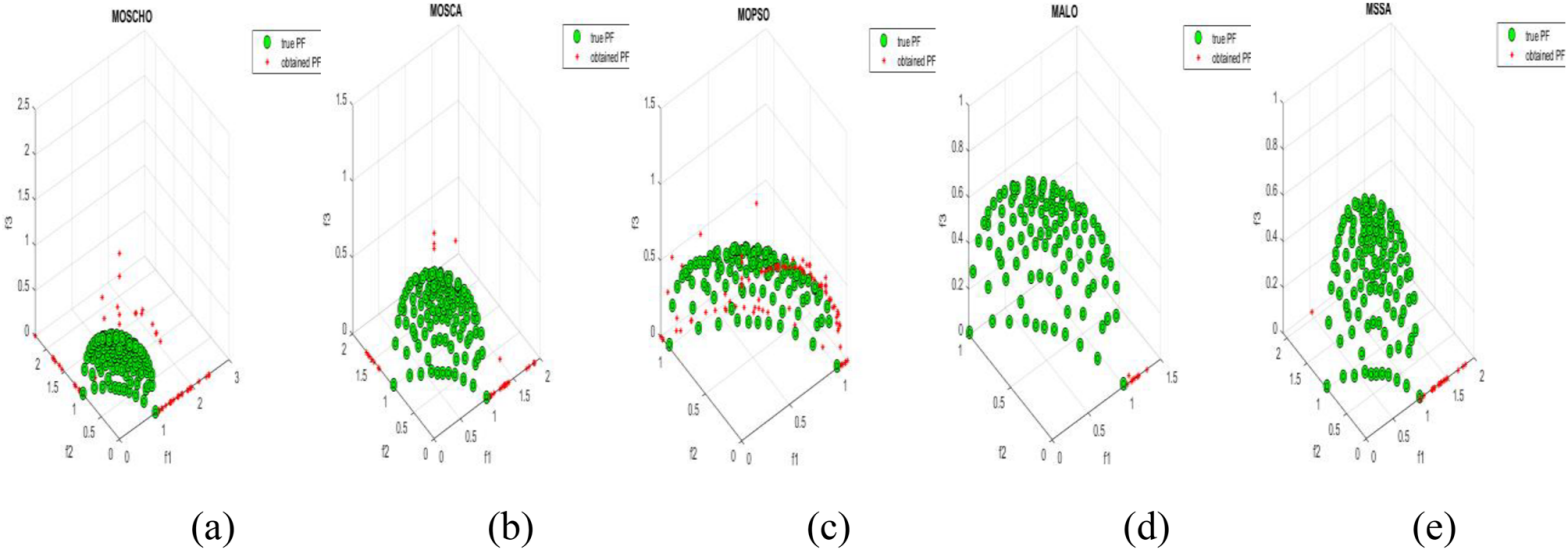
Obtained Pareto optimal set and true Pareto front of DTLZ1 problem. (**a**): MOSCHO, (**b**): MOSCA, (**c**): MOPSO, (**d**): MALO, (**e**): MSSA.

**Fig. 23:**
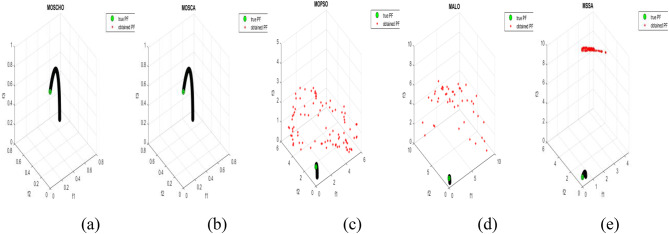
Obtained Pareto optimal set and true Pareto front of DTLZ4 problem. (**a**): MOSCHO, (**b**): MOSCA, (**c**): MOPSO, (**d**): MALO, (**e**): MSSA.

**Fig. 24:**
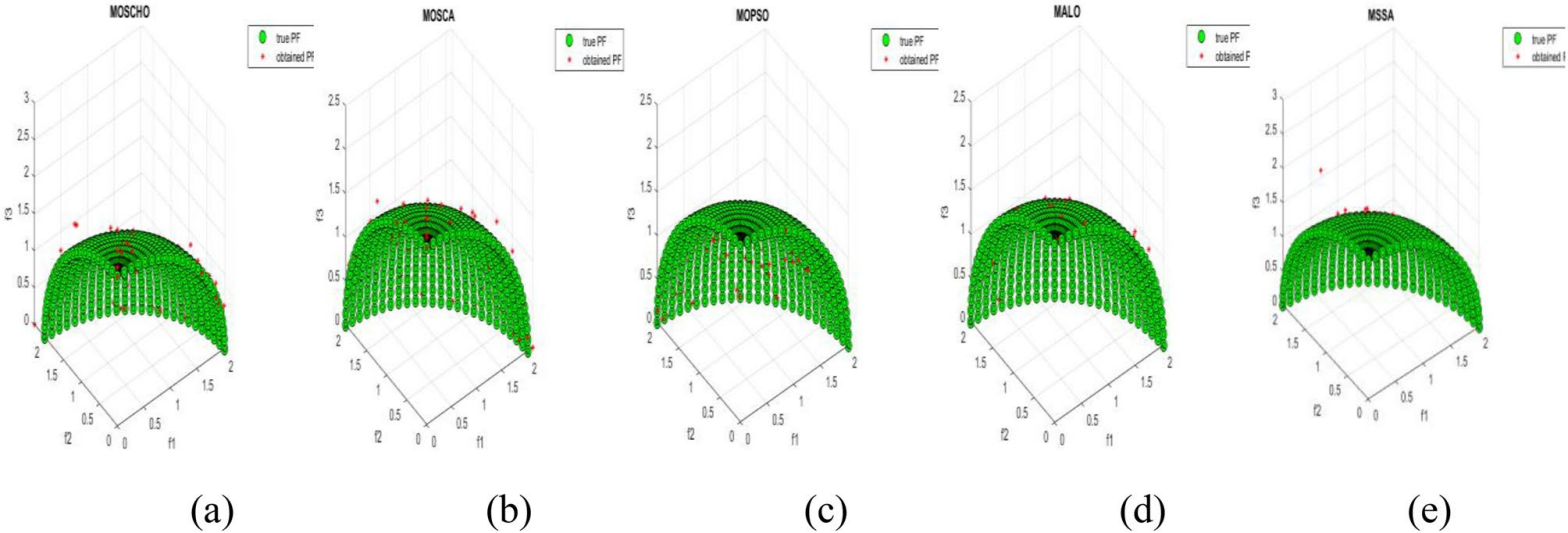
Obtained Pareto optimal set and true Pareto front of DTLZ6 problem. (**a**): MOSCHO, (**b**): MOSCA, (**c**): MOPSO, (**d**): MALO, (**e**): MSSA.

Figures 22 and 23, and 24 indicate true and obtained Pareto sets for the performance of MOSCHO and other compared algorithms, MOSCA, MPSO, MALO, and MSSA. In which MOSCHO has greater performance for both better distribution and good convergence of the obtained set compared to the true Pareto set for DTLZ4 and DTLZ6, and good performance for DTLZ1.

Where DTLZ1, DTLZ4, and DTLZ6 have concave geometries, and they are processed for three objectives. MOSCHO achieves good coverage and good convergence because of balanced integrated characteristics. The switch mechanism applies a good balance between exploration and exploitation phases, besides the difference between global solutions and memorized best local solutions to resize the search space to uniformly distribute the obtained solutions and update the next penetration of populations towards the global optimal solution, as shown in the Results of well-known problems (SRN, welded beam design problem).

#### Analysis results of the constrained benchmark function SRN, and the constrained welded beam design application

MOSCHO algorithm and other chosen algorithms are also tested in constrained applications, such as SRN and welded beam design applications. Design problems are the most challenging problems. Appendix B includes these two problems and all their constraints. The purpose of the SRN problem is to minimize two objectives, F1 and F2, simultaneously, which have two constraints. The purpose of the welded beam design problem is to minimize two objectives: end deflection and cost of the beam, which has four constraints. For testing MOSCHO and other chosen algorithms, the process is repeated fifteen times to make the results more consistent and more reliable.

**Table 9:** Statistical results of GD performance of the constrained test applications.

	GD	MALO	MSSA	MOPSO	MHS	MOSCA	MSCHO
SRN	Av	90.769	78.590	120.871	102.066	**67.428**	108.031
	SD	44.807	35.651	47.363	43.447	**28.022**	50.464
	WRT	-	-	+	+	+	
Welded beam	Av	11.284	14.480	9.366	13.802	10.462	**7.843**
	SD	6.925	4.925	3.410	4.319	4.183	**3.256**
	WRT	+	+	-	+	+	
W^+^/W^−^		89/47	112/35	62/41	150/0	150/0	

Table 9 shows the statistical results of the GD performance metric. MOSCHO has better performance for the welded beam design problem. But MOSCA has better performance for the SRN problem. Where SRN is a problem with only two constraints, and not as complicated as the welded beam application of four constraints. And MOSCHO obtains fast convergence at only 100 iterations for a complicated application.

The integrated characteristics of MOSCHO prove its convergence priority through the GD metric in welded beam engineering design application. And WRT represents positive differences more than negative differences because the best value of the GD metric is the minimum value. So, these results confirm MOSCHO’s ability to achieve the fastest convergence for complicated design engineering problems, as in the welded beam design application.

**Table 10:** Statistical results of ∆ performance of the constrained test applications.

	∆	NSGAII	MALO	MSSA	MWO	MHS	MOSCA	MSCHO
SRN	Av	0.413	1.477	0.830	0.716	0.761	0.868	**0.631**
	SD	0.026	0.062	0.059	0.050	0.060	0.128	**0.045**
	WRT	-	+	+	+	-	-	
Welded beam	Av	**0.623**	1.541	1.193	0.950	0.934	0.800	0.995
	SD	0.096	0.076	0.087	0.181	**0.059**	0.072	0.094
	WRT	-	+	+	-	-	-	
W^+^/W^−^		87/0	240/0	239/0	120/42	0/47	0/47	

Table 10 shows the statistical results of the diversity performance (∆) metric. MOSCHO has better performance for the SRN problem. But NSGAII and MOHS have better performance for the welded beam design problem. And WRT results confirm that the positive results are more than the negative results for the ∆ metric, the minimum value is the best. The Great diversity of obtained non-dominated solutions illustrated through the MOSCHO ∆ metric confirms the best distribution of the MOSCHO algorithm at 100 iterations for not complicated problems. And MOSCHO has good diversity for more complex engineering design applications. The ∆ metric’s results prove MOSCHO’s best coverage as a result of the best combination of MOSCHO characteristics.

**Fig. 25:**
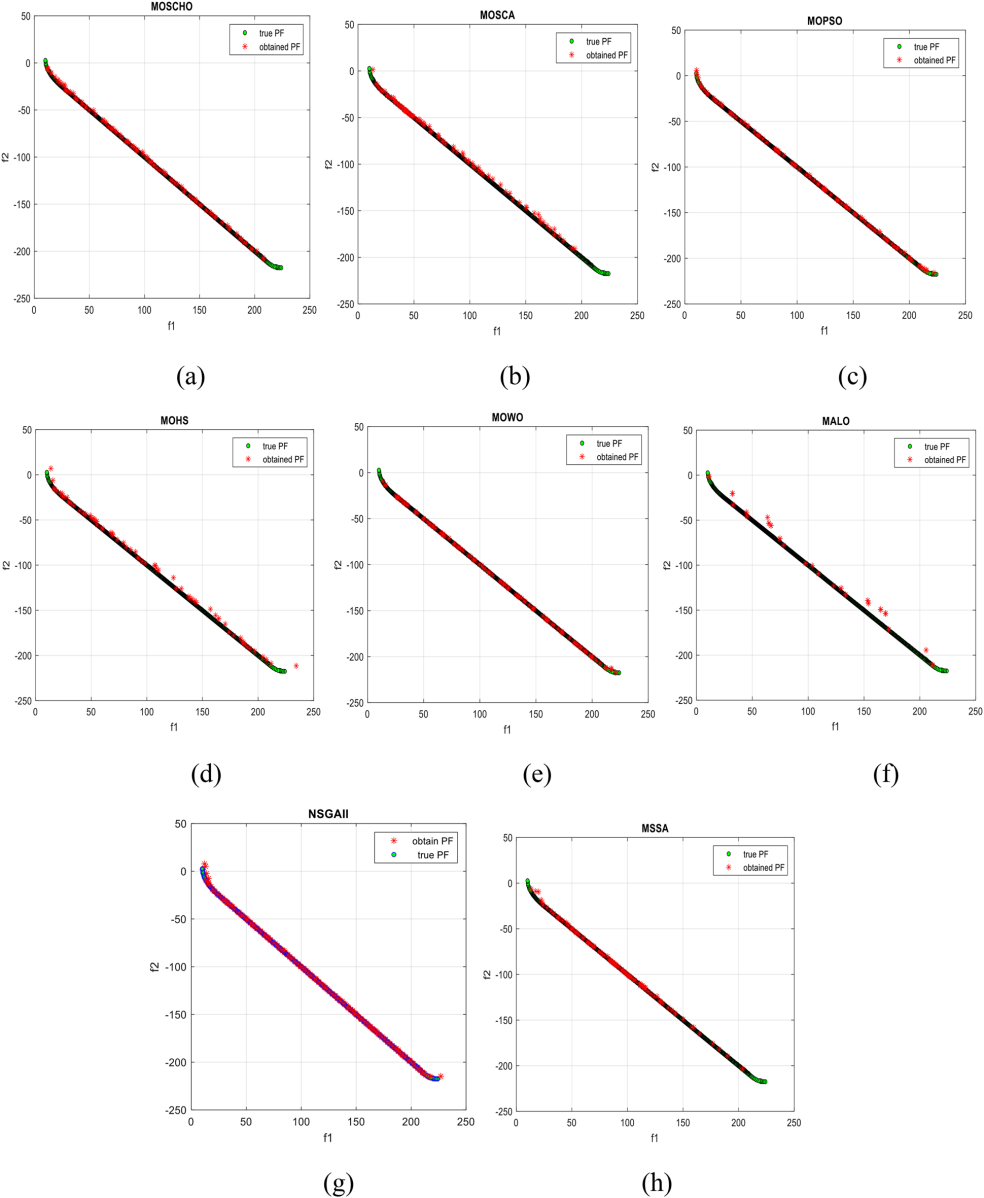
Obtained Pareto optimal set and true Pareto front of SRN problem. (**a**): MOSCHO, (**b**): MOSCA, (**c**): MOPSO, (**d**): MOHS, (**e**): MOWO, (**f**): MALO, (**g**): NSGAII, (**h**): MSSA.

Figure 25 indicates true and obtained Pareto sets for the performance of MOSCHO and other compared algorithms, MOSCA, MPSO, MHS, MWO, MALO, NSGAII, and MSSA. MOSCHO has better coverage and also a better convergence for the SRN problem due to its superior value of ∆ and GD metrics’ results. In which MOSCHO has great performance and great distribution of the obtained sets for the SRN problem compared to the true Pareto front set. The great performance confirms the superior benefits of combined characteristics for a constrained problem, such as bounded search based on global and memorized optimal solutions, and a switching mechanism between several phases. As illustrated in Tables 9, 10, 11 and 12. While the ER and SCC metrics’ results confirm the superior performance of MOSCHO.

**Table 11:** Statistical results of ER performance of the constrained test applications:.

	ER	NSGAII	MSSA	MOPSO	MWO	MHS	MOSCA	MSCHO
SRN	Av	0.9983	1	0.9990	1	0.9988	1	**0.9983**
	SD	0.0040	0	0	0.0030	0.0028	0	**0.0024**
	WRT	-	-	-	-	-	-	
Welded beam	Av	0.4990	0.500	**0.4986**	0.4988	0.4952	0.5000	**0.4986**
	SD	0.0020	**0**	0.0024	0.0100	0.0023	**0**	0.0027
	WRT	-	-	-	-	-	-	
W^+^/W^−^		0/5.2	0/5.5	0/5.1	0/5.7	0/4.8	0/5	

Table 11 shows statistical results of the error ratio (ER) performance metric of the constrained applications: SRN and the welded beam design problem. Where MOSCHO has better performance for both applications, SRN and welded beam design problems. Those two problems have the most solutions of the obtained PF that exist on the true PF. The ER results confirm the best performance (coverage and convergence) of MOSCHO’s success performance indicator of constrained simple and complicated problems as a result of MOSCHO’s good convergence and diversity of combined characteristics.

**Table 12:** Statistical results of SCC performance of the constrained test applications:.

	SCC	MALO	MSSA	MOPSO	MWO	MOSCA	MSCHO
SRN	Av	0.0666	0	0.2000	0	0	**0.3333**
	SD	0.2581	0	0	0.3518	0	**0.4879**
	WRT	-	-	-	-	-	
Welded beam	Av	**99.6666**	30.2666	91	85.4000	25.2666	78.5333
	SD	1.5886	9.8739	13.1246	3.8889	4.7878	**15.0184**
	WRT	+	-	+	-	+	
W^+^/W^−^		5.1/0	0/6.8	3.6/0	0/6.8	2.9/0	

Table 12 shows statistical results of the success counting (SCC) performance metric. MOSCHO has better performance for both applications, SRN and welded beam design problems. Where most of MOSCHO’s obtained PF are exist on the true PF of both simple and complex constrained problems. The SCC results illustrate MOSCHO’s best success performance because of the resulting great convergence and great distribution.

From Tables 1, 2, 3, 4, 5, 6, 7, 8, 9, 10, 11 and 12, MOSCHO has an excellent performance for some benchmark functions, such as ZDT3 and ZDT4 from ZDT problems. MOSCHO has better results for performance metrics for all CEC2009 for some metrics of each function. But for CEC2020, MOSCHO has an excellent performance for MMF14 and has superior values for more metrics of all CEC2020 except MMF1 and MMF2. MOSCHO has a better performance for DTLZ1, DTL4, and DTLZ6. however, MOSCHO has a better performance for GD, ∆, ER, and SCC metrics of the SRN constrained problem. MOSCHO has better performance for ER and SCC metrics of the welded beam design problem. These results confirm MOSCHO’s ability for complicated constrained engineering design problems.

**Fig. 26:**
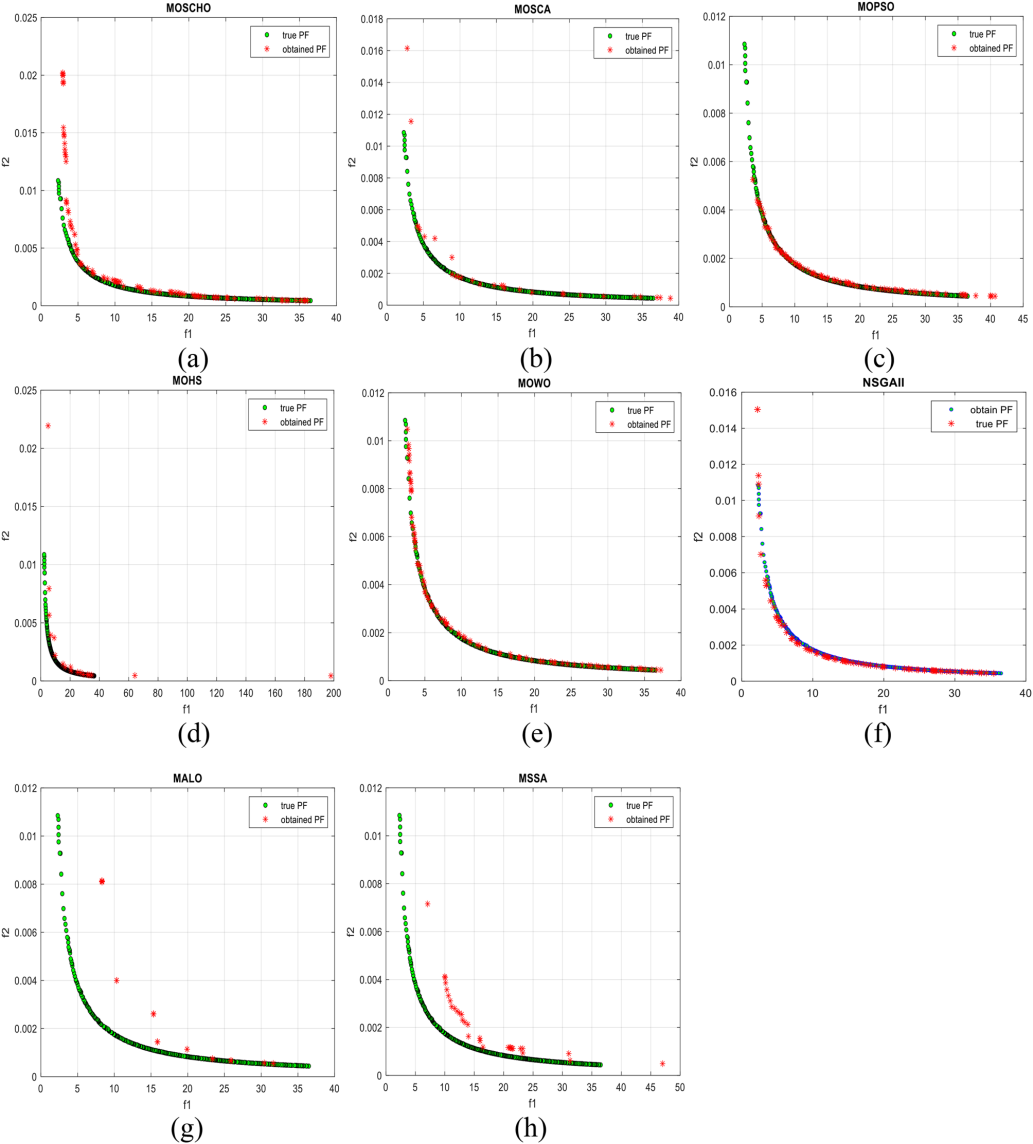
Obtained Pareto optimal set and true Pareto front of welded beam design problem. (**a**): MOSCHO, (**b**): MOSCA, (**c**): MOPSO, (**d**): MOHS, (**e**): MOWO, (**f**): MALO, (**g**): NSGAII, (**h**): MSSA.

Figure 26 indicates true and obtained Pareto sets for the performance of MOSCHO and other compared algorithms, MOSCA, MPSO, MHS, MWO, NSGAII, MALO, and MSSA. In which MOSCHO has excellent convergence and excellent distribution of the obtained set compared to the true Pareto set of the welded beam design application. Besides these figures, Tables 9, 10, 11 and 12 confirm MOSCHO’s performance superiority as a result of the integrated methods proposed for this experiment. Tables 11 and 12 confirm that the best number of obtained PF exists on the true PF achieved by the MOSCHO algorithm.

#### Analysis results of modern for real-world design problems

MOSCHO algorithm and other chosen algorithms are also tested in real-world applications, such as Optimal Power Flow^66^ and Optimal Setting of Droop Controller^67^ design electrical applications. Design problems are the most challenging problems. Appendix C includes details of these two problems. The purpose of the Optimal Power Flow problem is to minimize four objectives, F1, F2, F3, and F4, simultaneously. The purpose of the Optimal Setting of Droop Controller design problem is to minimize three objectives. For testing MOSCHO and other chosen algorithms, the process is repeated fifteen times to make the results more consistent and more reliable.

**Table 13:** Statistical results of the GD performance metric of the real-world test applications:.

	GD	NSGAII	MALO	MSSA	MOPSO	MOSCA	MOSCHO
Optimal power flow	Av	118.905	114.095	34.676	124.504	13.196	**9.870**
	SD	9.903	12.171	5.597	16.308	5.042	**3.062**
	WRT	+	+	+	+	+	
Optimal setting of droop controller	Av	1116.884	765.333	1070.041	**673.081**	972.647	1206.0392
	SD	**53.552**	140.838	207.817	118.609	148.997	178.249
	WRT	-	-	-	+	+	
W^+^/W^−^		120/30	120/0	120/34	206/0	206/0	

Table 13 shows the statistical results of the GD performance metric. MOSCHO has better performance for the Optimal Power Flow design problem. But MOPSO and NSGAII have better performance for the Optimal Setting of the Droop controller problem. The Optimal Power Flow design problem is a problem with only four objectives, and is more complicated than the Optimal Power Flow of only three objectives. And MOSCHO obtains fast convergence at only 100 iterations for a complicated application.

The integrated characteristics of MOSCHO prove its convergence priority through the GD metric in the Optimal Power Flow engineering design application. And WRT represents positive differences for all tested algorithms for the Optimal Power Flow design problem and more positive differences than negative differences for the Optimal Setting of Droop controller design problem, because the best value of the GD metric is the minimum value. So, these results confirm MOSCHO’s ability to achieve the fastest convergence for complicated design engineering problems, as in the Optimal Power Flow design application.

**Fig. 27:**
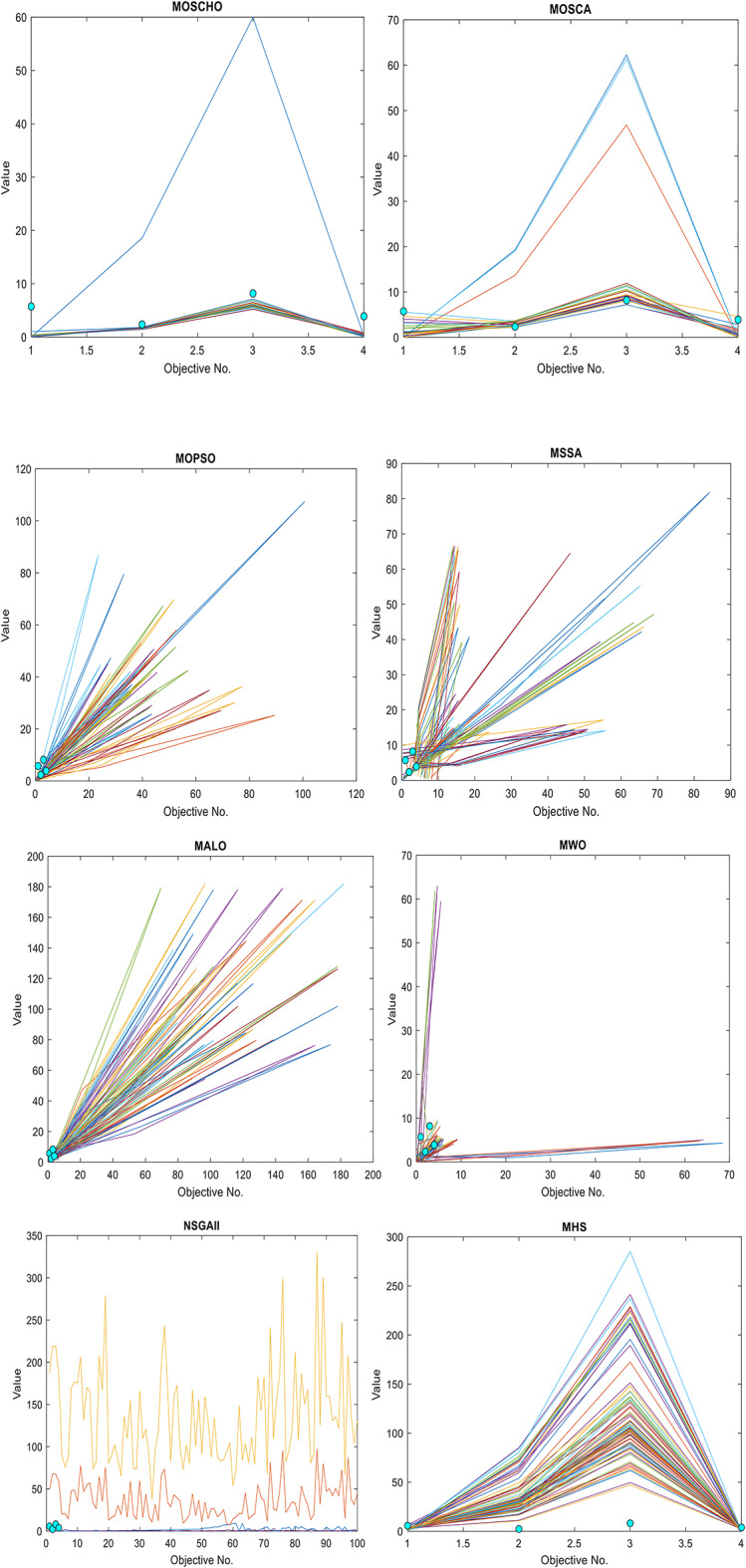
Obtained Pareto optimal set and true Pareto front of the optimal power flow design problem.

Figure 27 indicates the best comprise solution(BCS) and obtained Pareto sets for the performance of MOSCHO and other compared algorithms, MOSCA, MPSO, MHS, MWO, NSGAII, MALO, and MSSA. In which MOSCHO has better convergence and better distribution of the obtained set compared to the BCS of the Optimal Power Flow design application. But NSGAII handles objectives as separate subproblems. Besides these figures, Tables 14, 15 and 16 confirm MOSCHO’s performance superiority as a result of the integrated methods proposed for this experiment.

**Table 14:** Statistical results of IGD performance of the real-world test applications:.

	IGD	NSGAII	MSSA	MWO	MHS	MOPSO	MOSCHO
Optimal power flow	Av	93.680	25.244	32.317	7.290	73.382	**5.861**
	SD	25.709	15.322	4.839	20.188	23.648	**2.417**
	WRT	+	+	+	+	+	
Optimal setting of droop controller	Av	1006.622	717.727	949.029	1219.689	**446.062**	819.277
	SD	443.745	502.918	398.381	**312.204**	402.364	423.216
	WRT	+	-	+	-	+	
W^+^/W^−^		202/0	115/53	167/0	120/16	120/70	

Table 14 shows the statistical results of the IGD performance metric. MOSCHO has better performance for the Optimal Power Flow design problem. But MOPSO and MHS has better performance for the Optimal Setting of Droop controller design problem. The Optimal Power Flow design problem is a problem with only four objectives, and is more complicated than the Optimal Power Flow of only three objectives. And MOSCHO obtains fast convergence at only 100 iterations for a complicated application.

The integrated characteristics of MOSCHO prove its convergence priority through the GD metric in the Optimal Power Flow engineering design application. And WRT represents positive differences for all tested algorithms for the Optimal Power Flow design problem and more positive differences than negative differences for the Optimal Setting of Droop controller design problem, because the best value of the GD metric is the minimum value. So, these results confirm MOSCHO’s ability to achieve the fastest convergence for complicated design engineering problems, as in the Optimal Power Flow design application.

**Fig. 28:**
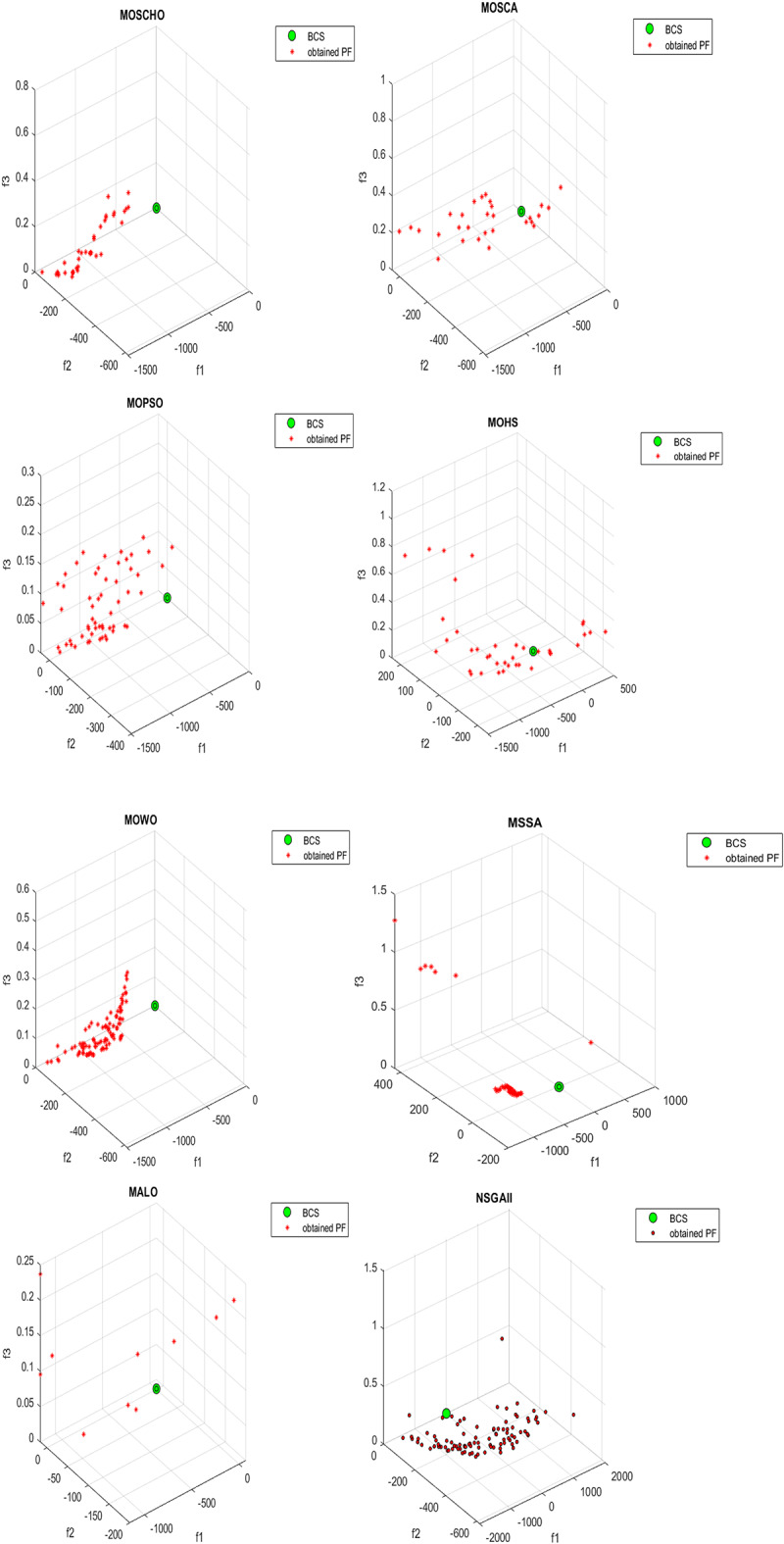
Obtained Pareto optimal set and true pareto front of the optimal setting of droop controller design problem.

Figure 28 indicates best comprise solution(BCS) and obtained Pareto sets for the performance of MOSCHO and other compared algorithms, MOSCA, MPSO, MHS, MWO, NSGAII, MALO, and MSSA. In which MOSCHO has better convergence and better distribution of the obtained set compared to the BCS of the Optimal Setting of Droop controller design application. Besides these figures, Tables 14, 15 and 16 confirm MOSCHO’s performance superiority as a result of the integrated methods proposed for this experiment.

**Table 15:** Statistical results of spacing performance of the real-world test applications:.

	S	NSGAII	MALO	MHS	MOPSO	MOSCA	MOSCHO
Optimal power flow	Av	11.409	10.730	3.0175	13.119	5.130	**2.657**
	SD	3.242	3.735	2.883	3.064	2.150	**1.848**
	WRT	+	+	+	+	+	
Optimal setting of droop controller	Av	232.921	111.360	**91.549**	131.046	151.266	116.415
	SD	30.641	40.088	29.769	**21.879**	40.197	51.563
	WRT	+	-	+	-	-	
W^+^/W^−^		240/0	120/54	197/0	105/7	105/7	

Table 15 illustrates the statistical results of the spacing performance metric (S) of modern real-world electrical applications. From metric spacing’s results, MOSCHO has the best performance of the Optimal Power Flow problem, but MHS and MOPSO have the best performance for the Optimal Setting of Droop controller.

While WRT approves that MOSCHO has differences of more than 5% differential, as in Table 13. For the spacing metric, the best value is the minimum value, where MOSCHO has 100% positive differences in the Optimal Power Flow problem. compared other tested algorithms, up to 30% positive differences in two algorithms compared to other tested algorithms.

As a result of a balance between exploration phases and exploitation phases and bounded search to resize the search space based on both the global optimal solution and memorized global solutions, MOSCHO has a great distribution of obtained Pareto optimal sets, which refers to good coverage of the algorithm.

### Parameters sensitivity analysis

To extend SCHO for handling multiple objective problems, many parameters need to be tuned to obtain good performance. MOSCHO’s parameter sensitivity is analyzed for 100 iterations and 100 candidate solutions for both population and repository to reach the best values.

For the original SCHO algorithm, the sensitivity parameters, such as A, ct, u, m, n, and Ꜫ, are tuned to perform SCHO for multi-objective problems. ZDT1, a multi-objective problem used to tune SCHO parameters for multi-objective optimization problems. Due to randomization, processing is repeated three times for the sensitivity parameters for GD and IGD metrics.

Parameter (A) is responsible for switching between exploration and exploitation optimization methods. Table 16 shows the sensitivity analysis of parameter (A) for various values, where the best value is 3.

**Table 16:** Average of the GD and IGD values on ZDT for A & Ct parameters.

A	GD	IGD	ct	GD	IGD
0.5	0.4929	0.40	0.5	0.679	0.4388
1	0.2433	0.3041	1	0.6811	0.417
1.5	0.4985	0.3075	1.5	0.5820	0.3483
2	0.4962	0.2845	2	0.3743	0.2254
2.5	0.5615	0.3740	2.5	0.5984	0.5941
**3**	**0.1501**	**0.0803**	3	0.5005	0.2801
3.5	0.4947	0.4152	3.5	0.2079	0.4115
4	0.4986	0.4173	4	0.5434	0.4584
4.5	0.166	0.0884	**4.5**	**0.147**	**0.1183**
5	0.2156	0.1171	5	0.4623	0.2972

The parameter (ct) is responsible for switching between the two phases of both exploration and exploitation methods. Table 16 summarizes tuning values for the parameter, where the best value is 4.5, which produces the best GD and IGD. The parameters (m) and (u) are responsible for controlling the accuracy of the 1 st phase of exploration and exploitation, which have values for best GD and IGD equal 0.1 and 0.6, as shown in Table 17. The parameters (n) and (Ꜫ) control the accuracy of the 2nd phase of exploration, while parameter (n) controls the 2nd phase exploitation only. The best values of both n and Ꜫ equal 0.7 and 0.003, respectively, which achieve the best GD and IGD values as shown in Table 18.

**Table 17:** Average of the GD and IGD values on ZDT for m & u parameters.

m	GD	IGD	u	GD	IGD
**0.1**	**0.0593**	**0.0473**	0.1	0.3576	0.1947
0.2	0.4952	0.3445	0.2	0.5507	0.3624
0.3	0.6436	0.4919	0.3	0.4050	0.5793
0.4	0.5627	0.5019	0.4	0.4288	0.2159
0.5	0.684	0.5369	0.5	0.4344	0.2651
0.6	0.6029	0.4005	**0.6**	**0.0154**	**0.088**
0.7	0.4323	0.2519	0.7	0.3869	0.4011
0.8	0.4404	0.2346	0.8	0.370	0.2281
0.9	0.5716	0.4121	0.9	0.5982	0.5511
1	0.2958	0.4549	1	0.4995	0.395

**Table 18:** Average of the GD and IGD values on ZDT for n & Ꜫ parameter.

*n*	GD	IGD	Ꜫ	GD	IGD
0.1	0.5046	0.2872	0.0001	0.4132	0.2514
0.2	0.3507	0.2663	0.0003	0.4346	0.2604
0.3	0.3768	0.1982	0.0005	0.4734	0.2555
0.4	0.3819	0.207	0.0008	0.5433	0.3261
0.5	0.3087	0.1627	0.001	0.5281	0.4920
0.6	0.3909	0.4573	0.002	0.393	0.2474
**0.7**	**0.1378**	**0.0724**	**0.003**	**0.1125**	**0.0593**
0.8	0.4081	0.2366	0.004	0.6823	0.6007
0.9	0.5216	0.3199	0.006	0.5288	0.3424
1	0.3765	0.2451	0.008	0.6262	0.3818

While parameters of SCHO are responsible for achieving a balance between exploration and exploitation methods, as mentioned before in this section. But the following tests are used to tune the parameters of the multi-objective main methods.

The main parameters of the MOSCHO algorithm are nGrid, α, β, γ, and the mutation parameter (mu), which control the proposed method for this experiment. Where nGrid and α parameters for the hyper-cube method, nGrid represents the number of candidate solutions for each hyper-cube, and α is an inflation rate that controls the nGrid parameter. While the β parameter controls the leader selection method, the γ parameter controls the deletion selection method. The mu parameter is a factor for the accepted percentage of the new candidate solution’s position obtained. As shown in Tables 19 and 20, and 21, for best performance, nGrid has a value equal to 25, α has a value equal to 0.3, β has a value equal to 3, γ has a value equal to 1.5, and mu has a value equal to 0.6.

**Table 19:** GD and IGD of some benchmark functions for the nGrid parameter.

nGrid	ZDT1		UF4		MMF11		MMF14	
	GD	IGD	GD	IGD	GD	IGD	GD	IGD
3	0.672	0.363	0.148	0.087	3.225	3.264	31.481	2.0424
5	0.750	0.322	0.598	0.341	2.973	2.916	34.01	12.516
7	0.501	0.379	0.504	0.412	2.779	2.876	33.805	17.628
10	0.441	0.292	0.693	0.349	2.871	2.668	23.403	2.980
12	0.358	0.210	0.596	0.431	2.831	2.905	24.003	3.125
15	0.160	0.083	0.400	0.250	2.651	2.705	34.670	11.826
18	0.056	0.031	0.726	0.351	3.609	3.701	33.697	3.168
20	0.025	0.018	0.487	0.290	3.115	2.913	39.667	47.540
**25**	**0.065**	**0.033**	**0.458**	**0.295**	**2.499**	**2.197**	**13.148**	**12.166**
30	0.280	0.166	0.642	0.531	2.790	3.059	35.332	35.976
35	0.067	0.037	0.352	0.201	3.789	3.890	38.861	51.629
40	0.223	0.124	0.670	0.453	2.575	2.610	23.834	32.617
45	0.207	0.105	0.765	0.490	3.758	3.910	20.235	22.870
50	0.139	0.073	0.498	0.250	3.449	3.281	31.496	2.459

**Table 20:** GD and IGD of some benchmark functions for α & β parameters.

α	ZDT1		MMF14		β	ZDT1		MMF14	
	GD	IGD	GD	IGD		GD	IGD	GD	IGD
0.1	0.4689	0.2792	33.2841	2.2165	0.0	0.5761	0.2891	10.6759	0.7047
0.2	0.6753	0.6122	37.2084	18.2747	0.1	0.3622	0.2529	29.0515	6.5167
**0.3**	**0.1376**	**0.0699**	**19.4658**	**5.4757**	0.3	0.0250	0.0136	27.6436	10.2514
0.4	0.6572	0.3205	24.3087	1.9374	0.5	0.0194	0.0807	29.2992	23.3053
0.5	0.3499	0.1808	31.5078	31.0257	0.8	0.0884	0.0467	36.9314	6.9045
0.6	0.1025	0.0558	30.9175	32.1511	1	0.5423	0.3382	31.1989	29.0433
0.7	0.7244	0.3579	23.1939	8.0271	1.5	0.0223	0.0364	32.0583	20.1494
0.8	0.6309	0.4793	29.5162	2.1466	2	0.2488	0.1493	23.3814	12.0874
0.9	0.4145	0.1874	21.2141	7.4850	2.5	0.1335	0.0729	23.3128	4.6371
1	0.7064	0.4996	36.8856	41.7104	**3**	**0.0352**	**0.0567**	**26.3112**	**7.7072**

**Table 21:** GD and IGD of some benchmark functions for γ & mu parameters.

γ	ZDT1		MMF14		mu	ZDT1		MMF14	
	GD	IGD	GD	IGD		GD	IGD	GD	IGD
0.0	0.1477	0.8402	27.6642	26.1568	0.1	0.0607	0.0298	29.9541	2.4089
0.1	0.3509	0.2043	26.7134	15.1444	0.2	0.1367	0.2043	33.2200	20.1184
0.3	0.6947	0.8096	29.9935	3.2762	0.3	0.6956	0.6831	21.6186	3.00600
0.5	0.3139	0.1727	34.0592	19.4727	0.4	0.1002	0.0566	28.2823	0.18610
0.8	0.3493	0.2744	27.6367	14.5821	0.5	0.5283	0.2844	28.2059	30.2227
1	0.1330	0.0710	30.514	19.4554	**0.6**	**0.0442**	**0.0229**	**11.1360**	**3.66990**
**1.5**	**0.0316**	**0.0174**	**2.2870**	**1.8761**	0.7	0.6525	0.5089	33.6887	44.7560
2	0.4191	0.2004	22.4622	10.0857	0.8	0.0455	0.0242	26.6603	2.79610
2.5	0.5873	0.4444	27.5850	9.89280	0.9	0.7291	0.3794	21.4594	13.9179

After tuning MOSCHO parameters, all parameters setting of all algorithms are aggregated in Table 22.

**Table 22:** All parameter settings of all algorithms.

parameter	NSGAII	MALO	MSSA	MHS	MWO	MOPSO	MOSCA	MOSCHO
Population size	100	100	100	100	100	100	100	100
Repository size	100	100	100	100	100	100	100	100
nGrid	-	25	25	25	25	25	25	25
α	-	0.3	0.3	0.3	0.3	0.3	0.3	0.3
β	-	3	3	3	3	3	3	3
γ	-	1.5	1.5	1.5	1.5	1.5	1.5	1.5
mu	1/D	0.6	0.6	0.6	0.6	0.6	0.6	0.6
A	3	-	-	-	-	-	-	-
ct	**4.5**	-	-	-	-	-	-	-
m	0.1	-	-	-	-	-	-	-
u	0.6	-	-	-	-	-	-	-
n	0.7	-	-	-	-	-	-	-
Ꜫ	0.003	-	-	-	-	-	-	-
Crossover probability	0.9	-	-	-	-	-	-	-
Personal learning coefficient	1	-	-	-	-	-	-	-
Global learning coefficient	1.5	-	-	-	-	-	-	-
Inertia weight	0.4	-	-	-	-	-	-	-
Harmony memory considering rate	-	-	-	0.95	-	-	-	-
Pitch adjustment rate	-	-	-	0.3	-	-	-	-

Table 22 shows all the parameter settings of the used algorithms in this experiment. Each algorithm has specific parameters that affect its process while updating the new positions of the candidate solutions. Table 22 summarizes initial values for the parameters of all tested algorithms and the initial parameters for the experiment, such as 100 populations, 100 repositories, and a maximum of 100 iterations were used for each experiment. But the parameters nGrid, α, β, and γ that were added while expanding the multi-objective techniques are used for all algorithms except NSGAII.

## Limitations of moscho’s proposed method

MOSCHO’s proposed method faces many main limitations:

- MOSCHO requires careful tuning of its parameters, and the tuning process requires time consumption, as indicated in Sect. 4.3. MOSCHO requires tuning more than ten parameters, which consumes more time for the parameter sensitivity process.
- The MOSCHO’s performance varies according to the problem characteristics and the problem environment and constraints, as indicated in Sect. 4.2 for both benchmark functions and real-world design applications. So the performance varies according to the testing environment.
- There are many difficulties faced in the multi-objective problems, in which maintaining equilibrium between all objectives of the problem is required to obtain an optimal solution of all objectives simultaneously and achieve balance between many objectives at the same time, as illustrated in the different problems’ results in Sect. 4.2.
- Handling multiple objectives makes the meta-heuristic algorithms more complicated.

## Conclusion and future works

The proposed MOSCHO algorithm provides excellent solutions for complex benchmark functions for all performance metrics in comparison with relevant works in the literature review. MOSCHO obtains good convergence with a perfect spreading simultaneously between a repository archived and a Pareto front set. where the memorized technique is based on saving the best local position of each candidate solution reached from the last iteration, and using the saved best solution of each population while updating the position at the current iteration. Memorized Multi-objective of Sinh-Cosh optimization algorithm has a better performance for convergence of repository comparing to known Pareto front set of each benchmark functions and real-world engineering design problems, as illustrated from tables of convergence, coverage, and performance success metrics’ results. MOSCHO achieves fast convergence. And MOSCHO has an excellent spreading of repository solutions on the Pareto front set due to spacing and diversity metrics. However, MOSCHO has better performance for the success performance indicator error ratio and success counting. It has better efficiency in successful performance indicator metrics for the tested real-world problem. But one of the MOSCHO’s main limitations, that MOSCHO requires multiple careful tuning of its parameters. The MOSCHO’s performance varies according to the problem characteristics and 

### Future works

- Extension of MOSCHO to many-objective problems (more than three objectives).
- Application of MOSCHO to more complex real-world engineering problems.
- Hybridization of MOSCHO with other algorithms to improve performance on specific problem types.
- Improvement of the algorithm’s constraints handling mechanism.
- Adaptation of MOSCHO for dynamic multi-objective optimization problems.

## Supplementary Information

Below is the link to the electronic supplementary material.

Supplementary Material 1

## Data Availability

All data generated or analyzed during this study are included in this published article.

## Code availability

For inquiries related to the code implementation and design, please contact the corresponding author.
