# Supplementary material for: A memorized multi-objective Sinh-Cosh optimizer for solving multi-objective engineering design problems
Source: Sci Rep. 2026 Jan 21;16:3039. doi: 10.1038/s41598-025-33789-8 (PMC12827340; doi:10.1038/s41598-025-33789-8)
Supplement: Supplementary file 1 — Supplementary Material 1 [file 41598_2025_33789_MOESM1_ESM.docx]

**Appendices for****A Memorized Multi-Objective Sinh-Cosh Optimizer for Solving Multi-Objective Engineering Design Problems**

# **Appendix A: Some non-constrained mathematical benchmark functions for both bi-objective and tri-objective functions.**

## **ZDT1 [59]:**

Minimize: $F_{1}\left( x \right)=x_{1}$

Minimize: $F_{2}\left( x \right)=g \left( 1- \sqrt{\frac{F_{1}}{g}} \right)$

Where: $g\left( x \right)=1+ \frac{9}{d-1}\sum_{i=2}^{d} x_{i}$ $x_{i}\in\left[ 0,1 \right]$

## **ZDT3 [59]:**

Minimize: $F_{1}\left( x \right)=x_{1}$

Minimize: $F_{2}\left( x \right)=g \left( 1- \sqrt{\frac{F_{1}}{g}}- \frac{F_{1}}{g}\sin\left( 10\pi F_{1} \right) \right)$

Where: $g\left( x \right)=1+ \frac{9}{d-1}\sum_{i=2}^{d} x_{1}$ $x_{i}\in\left[ 0,1 \right]$

## **UF4 [65]:**

Minimize: $F_{1}\left( x \right)=x_{1}+\frac{2}{\left| g_{1}\left( x \right) \right|} \sum_{i\in g_{1}} h\left( y_{i} \right)$

Minimize: $F_{2}\left( x \right)=1-{x_{1}}^{2}+\frac{2}{\left| g_{2}\left( x \right) \right|} \sum_{i\in g_{2}} h\left( y_{i} \right)$

Where: $g_{1}= \left\{ g | g is odd and 2 \leq g\leq n \right\}$

: $g_{2}= \left\{ g | g is even and 2 \leq g\leq n \right\}$

$y_{i}\left( x \right)=x_{j}-\sin\left( 6\pi x_{1}+ \frac{j\pi}{n} \right) j=2,\ldots.,n$

$h\left( t \right)=\frac{\left| t \right|}{1+ e^{2\left| t \right|}}$ $x_{i}\in\left[ 0,2 \right]$

## **MMF10 [61]:**

Minimize: $F_{1}\left( x \right)=x_{1}$

Minimize: $F_{2}\left( x \right)={\frac{g\left( x_{2} \right)}{x_{1}}}$

Where: $g\left( x \right)=2-\exp\left[ \left( \frac{x=0.2}{0.004} \right)^{2} \right]-0.8exp\left[ \left( \frac{x-0.6}{0.4} \right)^{2} \right]$

$x_{i}\in\left[ 0.1,1.1 \right]$

## **DTLZ1 [60] :**

Minimize: $F_{1}\left( x \right)=0.5\left( 1+g \right)x_{1}x_{2}$

Minimize: $F_{2}\left( x \right)=0.5\left( 1+g \right) x_{1}x_{2}$

Minimize: $F_{3}\left( x \right)=0.5\left( 1+g \right)\left( 1- x_{1} \right)$

Where: $g\left( x \right)= \sum_{i=3}^{d} \left( x_{i}-0.5 \right)^{2}$ $x_{i}\in\left[ 0,1 \right]$

## **DTLZ4 [60]:**

Minimize: $F_{1}\left( x \right)=\left( 1+g \right)\cos\left( {x_{1}}^{\pi}\left( \frac{\pi}{2} \right) \right)\cos\left( {x_{2}}^{\pi}\left( \frac{\pi}{2} \right) \right)$

Minimize: $F_{2}\left( x \right)=\left( 1+g \right)\cos\left( {x_{1}}^{\pi}\left( \frac{\pi}{2} \right) \right)\sin\left( {x_{2}}^{\pi}\left( \frac{\pi}{2} \right) \right)$

Minimize: $F_{3}\left( x \right)=\left( 1+g \right)\sin\left( {x_{1}}^{\pi}\left( \frac{\pi}{2} \right) \right)$

Where: $g\left( x \right)= \sum_{i=3}^{d} \left( x_{i}-0.5 \right)^{2}$ $x_{i}\in\left[ 0,1 \right]$

**Appendix B: constrained real-world problems**

**SRN**

SRN is a constrained real-world problem that has a continuous Pareto optimal front [62, 63].

Minimize: $F_{1}\left( x \right)=2+\left( x_{1}-2 \right)^{2}+\left( x_{2}-1 \right)^{2}$

Minimize: $F_{1}\left( x \right)={9x}_{1}\left( x_{2}-1 \right)^{2}$

Where: $g_{1}\left( x \right)={x_{1}}^{2}+ {x_{2}}^{2}-255$

$g_{2}\left( x \right)=x_{1}-3x_{2}+10$

$-20 \leq x_{1}\geq20 , -20 \leq x_{2}\geq20$

**Welded beam design problem**

Welded beam design is a constrained engineering problem that has two objectives: fabrication cost and beam deflection for equations F_1_ and F_2,_ respectively. It has four variables representing wild thickness, clamp bar length, bar height, and bar thickness represented for x_1_, x_2,_ x_3,_ and x_4,_ respectively [64].

Minimize: $F_{1}\left( x \right)=1.10471\times{x_{1}}^{2}\times x_{2} +0.04811\times x_{3}\times x_{4}\left( 14+x_{2} \right)$

Minimize: $F_{2}\left( x \right)=\frac{65856000}{30\times{10}^{6}\times x_{4}\times{x_{3}}^{3}}$

Where: $g_{1}\left( x \right)=\tau-13600$

$g_{2}\left( x \right)=\sigma-30000$

$g_{3}\left( x \right)=x_{1}- x_{4}$

$g_{4}\left( x \right)=6000-P$

$0.125 \leq x_{1}\geq5 , 0.1 \leq x_{2}\geq10$

$0.1 \leq x_{3}\geq10 , 0.125 \leq x_{4}\geq5$

Where: $q=6000\left( 14+\frac{x_{2}}{2} \right)$

$D=sqrt\left( \frac{{x_{2}}^{2}}{4}+\frac{x_{1}+{x_{3}}^{2}}{4} \right)$

J $=2 \left( \sqrt{2}x_{1}x_{2}\left( \frac{{x_{2}}^{2}}{12}+ \frac{\left( x_{1}+ x_{3} \right)^{2}}{4} \right) \right)$

$\alpha=\frac{6000}{\sqrt{2} {x_{1}x}_{2}}$

$\beta=Q \times\frac{D}{J}$


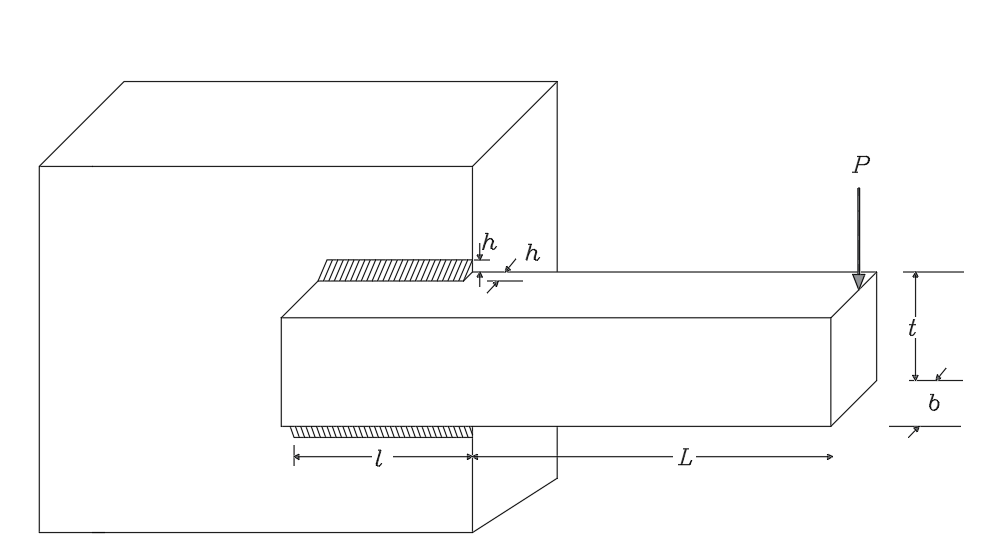


Figure 1: welded beam design application [64]

**Appendix C: real-world electrical engineering applications:**

## **The Optimal Power Flow [66]:**

It is one of the electrical applications that is optimized to minimize fuel cost, active and reactive power loss, and voltage deviation for microgrids. It has four objectives and thirty-four variables.

Minimize: $F_{1}\left( x \right)= \sum_{i=1}^{n} a_{1}+b_{1}P_{g}+ c_{1}{P_{g}}^{2}$

Minimize: $F_{2}\left( x \right)= real\left( V_{1}+conj (I_{1}) \right)+\sum_{i=2}^{14} real(S)$

Minimize: $F_{3}\left( x \right)=imag\left( V_{1}+conj (I_{1}) \right)+\sum_{i=2}^{14} imag(S)$

Minimize: $F_{3}\left( x \right)= \sum_{i=2}^{14} 1-\left( \left| V \right| \right)^{2}$

Where: $S=V\times conj(I)$

$$g=[ 1, 2 , 3 , 6 , 8]$$

$$a_{1}=[ 0 , 0 , 0 , 0 , 0]$$

$$b_{1}=[ 2 , 1.75 , 1 , 3.25 , 3]$$

$$c_{1}=[0.02 , 0.0175 , 0.0625 , 0.00834 , 0.025]$$

Where: -1$\leq x_{1}: x_{26}\geq1 , 0 \leq{x_{27} : x}_{34}\geq2$

## **The Optimal Setting of the Droop Controller [67]:**

It is one of the electrical applications that is optimized to minimize active and reactive power loss and voltage deviation for microgrids. It has three objectives and eighteen variables.

Minimize: $F_{1}\left( x \right)= \sum_{i=1}^{n} (P_{c}\left( 1-w \right)-P_{1}\left( \frac{\left| V \right|}{P_{5}} \right)^{P_{6}})$

Minimize: $F_{2}\left( x \right)= \sum_{i=1}^{n} \left( Q_{v}(1-\sqrt{({V_{r}}^{2}+{V_{m}}^{2})} \right)-Q_{1}\left( \frac{\left| V \right|}{Q_{5}} \right)^{Q_{6}}$

Minimize: $F_{3}\left( x \right)= \sum_{i=1}^{n} \left( 1-\left| V \right| \right)^{2}$

Where: $w=x_{11}$

$V_{r}=real(V)$

$V_{m}=imag(V)$

Where -1$\leq x_{1}: x_{10}\geq1 , 0 \leq x_{11}{: x}_{12}\geq2 , 0 \leq x_{13}: x_{18}\geq1$
